# Comparative Assessment of Crushed Glass Aggregate, Fly Ash, and Mining Tailings in Cementitious Composites and Shotcrete Applications

**DOI:** 10.3390/ma19194049

**Published:** 2026-09-22

**Authors:** Steven Perren, Ali Mirzaghorbanali, Hadi Nourizadeh, Paulomi (Polly) Burey, Kevin McDougall

**Affiliations:** 1 Centre for Future Materials (CFM), University of Southern Queensland, Toowoomba, QLD 4350, Australia; ali.mirzaghorbanali@unisq.edu.au (A.M.); hadi.nourizadeh@unisq.edu.au (H.N.); polly.burey@unisq.edu.au (P.B.); 2 School of Science, Engineering and Digital Technologies, University of Southern Queensland, Toowoomba, QLD 4350, Australia

**Keywords:** waste-derived materials, supplementary cementitious materials, cementitious composites, mining tailings, crushed glass aggregate

## Abstract

**Highlights:**

- Comparative review of CGA, fly ash, and mining tailings in cementitious composites.
- Mechanism-based assessment of hydration, microstructural development, and ITZ behaviour.
- Effects on rheology, mechanical performance, durability, and sustainability are synthesised.
- Critical research gaps identified for hybrid CGA-FA and tailings-FA systems.
- Research roadmap proposed for sustainable shotcrete and cementitious materials.

**Abstract:**

Waste-derived materials are increasingly being investigated as aggregate replacements and supplementary cementitious materials (SCMs) in cementitious composites due to their potential to improve resource efficiency while maintaining engineering performance. This review critically evaluates waste-derived materials used as aggregate replacements and SCMs, with particular emphasis on crushed glass aggregate (CGA), fly ash (FA), and mining tailings. Unlike previous reviews that focus primarily on individual waste streams, this study provides a comparative and mechanism-based assessment of these materials within a unified framework relevant to cementitious composites and shotcrete applications. Their influence on mechanical performance, durability, rheology, and microstructural development is assessed through the review of 78 publications, together with the governing mechanisms of packing effects, pozzolanic reactivity, and interfacial transition zone (ITZ) modification. Across the reviewed studies, CGA generally demonstrated favourable performance at moderate aggregate replacement levels (approximately 15–30%), FA commonly improved workability and durability at cement replacement levels of 10–20%, while mining tailings exhibited highly source-dependent behaviour, with performance strongly influenced by tailing type, mineralogy, processing history, and functional role within the cementitious system. Variability in physicochemical properties and processing methods contributed to inconsistent behaviour and limited direct comparison between studies. Critical knowledge gaps are identified, including long-term durability and hybrid CGA–FA and FBMT–FA systems. A comparative framework and research roadmap are proposed to support the development of durable and resource-efficient cementitious composites.

## 1. Introduction

Global waste generation is projected to increase substantially over the coming decades, creating significant challenges for environmental sustainability and resource management [1]. In Australia, large volumes of industrial and mining waste remain underutilised despite possessing properties suitable for construction applications and are often disposed of in landfill [2]. Although glass represents a relatively small fraction of the waste stream, large quantities remain unrecovered because of contamination and processing limitations [2,3,4]. Similarly, mining operations generate vast quantities of tailings, with approximately 620 Mt produced annually, the majority of which are deposited in long-term storage facilities rather than being reused [2].

Mining activities generate fine-grained waste materials during mineral extraction and processing, commonly referred to as tailings. These materials are typically pumped as slurry into tailings storage facilities (TSFs), which pose both environmental risks and opportunities for material reuse [5,6]. Failures of TSFs due to overtopping, mismanagement, or design deficiencies illustrate the need for safer and more sustainable waste strategies [7]. Regulatory compliance for tailings management alone costs the Australian mining industry approximately AUD 1.5 billion per year [8]. Both waste streams offer potential opportunities for incorporation into cementitious materials, reducing dependence on virgin resources while supporting circular economy principles [2,7]. Furthermore, the physical characteristics of CGA, FA, and mining tailings make them potential alternatives to conventional aggregate and cementitious constituents in concrete and shotcrete applications.

Shotcrete is widely used in mining, tunnelling, civil infrastructure, and structural rehabilitation due to its rapid placement, ability to conform to irregular surfaces, and suitability for ground support applications [9,10]. Owing to its high cementitious content and substantial fine aggregate requirements, shotcrete provides a particularly relevant platform for evaluating waste-derived materials as alternatives to natural aggregates and conventional supplementary cementitious materials.

The construction industry is one of the largest consumers of natural resources and a major contributor to environmental degradation, driven by increasing demand for cement, concrete, and natural aggregates. Global sand consumption alone exceeds 50 billion tonnes annually and is projected to rise significantly in the coming decades, raising concerns regarding resource depletion and ecological impacts [11,12]. The incorporation of waste-derived materials into cementitious systems presents a promising pathway to improve sustainability while maintaining engineering performance.

Several review studies have examined the use of individual waste-derived materials such as CGA, FA, and mining tailings in cementitious systems. However, existing reviews have largely focused on individual waste streams and conventional concrete applications, with limited comparative assessment of these materials within a common framework. In particular, direct comparisons of CGA, FA, and mining tailings with respect to their functional roles, hydration mechanisms, durability performance, and suitability for shotcrete applications remain limited. Consequently, the relative advantages, limitations, and practical readiness of these materials for shotcrete systems are not well established.

The performance of cementitious composites is governed by hydration reactions that form calcium silicate hydrate (C–S–H), calcium hydroxide (CH), ettringite (AFt), and monosulfoaluminate (AFm), which collectively influence pore structure development and ITZ characteristics [13,14,15,16]. Consequently, understanding how waste-derived materials influence these hydration processes is essential for evaluating their effects on fresh-state behaviour, mechanical performance, and long-term durability.

This review addresses that gap through a comparative, mechanism-informed assessment of CGA, FA, and mining tailings in cementitious composites, with a particular focus on shotcrete applications. The review evaluates material characteristics, fresh-state behaviour, mechanical performance, durability, hydration mechanisms, and microstructural development within a unified framework, while identifying key research gaps and future priorities for shotcrete and sustainable cementitious systems.

To enable a unified and systematic comparison, the reviewed materials are categorised based on their functional roles as (i) cement replacement materials, (ii) aggregate replacement materials, and (iii) hybrid systems incorporating multiple waste streams. This framework facilitates a comparative understanding of how waste-derived materials influence hydration, microstructure, and engineering performance. The methodology used to identify and screen the reviewed literature is described in the section Literature Selection Methodology. A total of 78 peer-reviewed studies were included in the final review. A comprehensive database of reviewed studies, including application type, replacement strategy, experimental design, and key findings, is provided in Supplementary Tables S1–S3.

### Literature Selection Methodology

Literature searches were conducted using Scopus, Web of Science, ScienceDirect, and Google Scholar between April 2024 and May 2026. Searches were conducted using combinations of the keywords “crushed glass aggregate”, “waste glass”, “fly ash”, “mining tailings”, “iron ore tailings”, “gold tailings”, “copper tailings”, “red mud”, “shotcrete”, “cementitious composites”, “concrete”, “supplementary cementitious materials”, “hydration”, “microstructure”, and “durability”. Representative search combinations included (“crushed glass aggregate” OR “waste glass”) AND concrete, (“fly ash”) AND shotcrete, and (“mining tailings” OR “iron ore tailings” OR “gold tailings” OR “copper tailings” OR “red mud”) AND cementitious composites.

Studies were initially screened based on title and abstract, followed by full-text review where required to determine relevance to aggregate replacement, cement replacement, fresh-state behaviour, mechanical performance, durability, hydration mechanisms, and microstructural development in cementitious systems. Additional relevant studies were identified through reference lists of key review papers and frequently cited publications. Peer-reviewed journal articles were prioritised, while conference papers and grey literature were considered only where directly relevant.

Studies were included if they:

i. Investigated CGA, FA, mining tailings, or hybrid waste-material systems in cementitious composites;
ii. Were available as full-text articles and contained original experimental data relevant to aggregate replacement, cement replacement, SCM applications, or hybrid systems.

Studies were excluded if they:

i. Focused on non-cementitious applications;
ii. Lacked sufficient technical detail to support comparative assessment;
iii. Were not relevant to the scope of cementitious composites or shotcrete applications.

A total of 78 publications were included and classified according to material type (CGA, FA, mining tailings, or hybrid systems), application category, replacement strategy, and investigated performance indicators. Publications directly reviewed and containing original experimental data were classified as “Primary Experimental Studies” and formed the basis of the comparative assessment. Review articles were used to identify broader research trends and relevant literature, while studies cited indirectly through other publications were classified as “Secondary Citations”. Secondary citations were included for contextual completeness but were not weighed equally to primary experimental studies during evidence synthesis. Publications were categorised according to the primary waste material investigated and further grouped based on functional role, including aggregate replacement, cement replacement, SCM application, or hybrid system incorporating multiple waste streams. Where publications investigated multiple waste materials or replacement strategies, they were assigned to all relevant categories for comparative assessment while being counted only once within the overall literature database.

Given the variability in mixture proportions, replacement strategies, curing conditions, material characteristics, and testing standards among the reviewed publications, a qualitative evidence appraisal approach was adopted. Greater emphasis was placed on findings consistently reported across multiple independent studies and supported by direct experimental measurements of fresh-state, mechanical, durability, hydration, or microstructural performance. Conversely, conclusions derived from isolated studies or limited datasets were interpreted with caution and identified as areas requiring further investigation. A complete database of reviewed studies is provided in Supplementary Tables S1–S3.

## 2. Waste-Derived Materials in Cementitious Composites

The incorporation of waste-derived materials into cementitious systems has gained increasing attention due to growing concerns regarding resource depletion, waste management, and the environmental impacts associated with the extraction and consumption of conventional construction materials [1,5,11,17]. The construction industry consumes substantial quantities of natural aggregates and cementitious materials, prompting increased interest in alternative resources capable of reducing environmental burdens while supporting sustainable development objectives.

Previous reviews have highlighted the feasibility of incorporating a wide range of waste-derived materials into cementitious systems, including concrete, mortar, and shotcrete, while demonstrating that material performance is strongly influenced by material characteristics, replacement strategies, and mixture design parameters [18,19,20,21,22,23,24,25]. Consequently, considerable research has been directed towards identifying waste materials that can partially or fully replace conventional aggregates and cementitious constituents without compromising engineering performance.

Among the various waste streams investigated, CGA, FA, and mining tailings have emerged as prominent candidates for construction applications owing to their availability, material characteristics, and potential for resource recovery [18,19,20,21,22,23,24,25]. Although these materials are frequently considered within the same sustainability framework, they fulfil distinct functional roles within cementitious systems.

CGA is primarily utilised as a replacement for natural fine aggregate owing to its high silica content and physical similarity to natural sand [23,25]. FA is commonly employed as a SCM due to its pozzolanic reactivity and ability to contribute to cementitious hydration processes [18]. In contrast, mining tailings represent a diverse material class that may function as aggregate replacements, cementitious constituents, or filler materials depending on their mineralogical composition, particle morphology, fineness, and processing history [19,20,21,22,23,24].

The suitability of these materials for cementitious applications is governed by factors such as particle size distribution, particle morphology, surface characteristics, chemical composition, and reactivity. Therefore, understanding their material characteristics, principal advantages, and inherent limitations is essential before evaluating their potential application in concrete, mortar, and shotcrete systems. The following sections provide a concise overview of CGA, FA, and mining tailings, focusing on their characteristics, advantages, and limitations.

### 2.1. Crushed Glass Aggregate (CGA)

CGA is one of the most widely investigated waste-derived materials for construction applications and is primarily utilised as a replacement for natural fine aggregate in cementitious systems [23,25,26]. It is produced from post-consumer and industrial waste glass streams, providing a potential pathway for diverting glass waste from landfill while reducing demand for natural sand resources [1,23].

CGA is predominantly composed of amorphous silica and exhibits chemical characteristics comparable to those of natural siliceous aggregates [23,25]. The physical properties of CGA depend on the source glass, crushing process, and grading employed. Crushed glass particles are typically angular in shape, possess smooth and impermeable surfaces, and exhibit negligible water absorption compared to natural sand [23]. Particle size distribution and surface texture vary considerably among recycled glass sources, contributing to differences in performance between studies.

The primary advantage of CGA is its potential to replace natural fine aggregate while simultaneously reducing waste disposal requirements and conserving natural resources [1,23]. The high silica content of glass makes it suitable for incorporation into cementitious systems, and finely divided glass may also exhibit pozzolanic behaviour when processed to sufficiently small particle sizes [23,25]. In addition, waste glass is widely available in many regions, making it an attractive candidate for sustainable construction applications [1].

Despite these benefits, several limitations restrict the widespread adoption of CGA. The angular morphology of crushed glass particles may reduce packing efficiency and affect fresh mixture behaviour, making performance dependent on particle grading and size distribution [23,27]. Variations in colour, composition, particle shape, and processing methods can also contribute to inconsistent performance between studies [23,25]. Furthermore, the high silica content of glass may introduce concerns regarding alkali–silica reaction (ASR) if appropriate mitigation measures are not considered [23,25,28]. The long-term behaviour of CGA-containing systems remains dependent on material characterisation, particle size distribution, mixture design, and compatibility with other cementitious constituents [23,25].

Overall, CGA represents a promising alternative to natural fine aggregate due to its availability, silica-rich composition, and potential environmental benefits. However, its successful utilisation requires careful consideration of particle characteristics, processing methods, and durability-related limitations.

### 2.2. Fly Ash (FA)

FA is one of the most widely used SCMs in concrete and shotcrete applications. It is a by-product generated during the combustion of pulverised coal in thermal power plants and has been extensively incorporated into cementitious systems as a partial replacement for Portland cement [18,29,30,31,32,33]. The widespread use of FA is primarily attributed to its availability, relatively low cost, and potential to utilise an industrial by-product that would otherwise require disposal.

The characteristics of FA are strongly influenced by the source of coal, combustion conditions, and particle fineness [18,30]. FA particles are generally spherical in shape and consist predominantly of silica, alumina, and varying amounts of calcium oxide [18]. Depending on its chemical composition, FA may exhibit either pozzolanic behaviour or a combination of pozzolanic and cementitious activity [18]. The spherical morphology and fine particle size of FA distinguish it from many other waste-derived materials used in cementitious systems and contribute to its widespread adoption as an SCM [18].

The primary advantages of FA are associated with its pozzolanic reactivity and established use in cementitious materials. FA can participate in secondary hydration reactions by consuming calcium hydroxide and forming additional calcium silicate hydrate (C–S–H), while simultaneously reducing Portland cement demand [18]. In addition, the spherical morphology of FA particles may enhance mixture flowability and reduce water demand compared with angular particles [18,29,30]. The extensive history of FA utilisation has also resulted in well-established standards and practical experience for its incorporation into concrete and shotcrete applications [18].

Despite these advantages, several limitations affect the use of FA in cementitious systems. The chemical composition, calcium content, fineness, and reactivity of FA vary depending on the source material and production process, leading to variability in behaviour [18]. The availability of FA is also becoming increasingly constrained in some regions due to the gradual reduction of coal-fired power generation [18]. Furthermore, FA generally exhibits lower early-age reactivity than Portland cement, meaning its effectiveness is highly dependent on particle fineness, replacement level, curing conditions, and mixture design [18,29,30,31,32]. Studies have also reported slower strength development and extended curing requirements at higher replacement levels, particularly under unfavourable curing conditions [29,31,32].

Overall, FA remains one of the most widely used waste-derived SCMs owing to its pozzolanic characteristics, industrial availability, and established use in cementitious systems. However, variations in material quality, declining availability in some regions, and dependence on mixture design and curing conditions remain important considerations for its successful utilisation.

### 2.3. Mining Tailings

Mining tailings are among the largest industrial waste streams generated worldwide and are produced during the extraction and beneficiation of mineral resources. Large volumes of tailings are commonly stored in tailings storage facilities (TSFs), creating environmental, economic, and land-use challenges for the mining industry [5,6,7,34]. Consequently, increasing attention has been directed towards identifying value-added applications for tailings in construction materials, including concrete, mortar, and shotcrete [19,20,21,22,24].

Unlike CGA and FA, mining tailings do not represent a single material but rather a diverse class of waste products whose characteristics depend on the parent ore, beneficiation process, and storage conditions. Tailings may originate from iron ore, gold, copper, coal, bauxite, and numerous other mining operations. As a result, considerable variability exists in particle size distribution, particle morphology, specific gravity, mineralogical composition, and chemical composition. Depending on these characteristics, mining tailings may be incorporated into cementitious systems as fine aggregate replacements, SCMs, filler materials, or combinations thereof [19,20,21,22,24].

Mining tailings commonly contain varying proportions of silica, alumina, calcium-bearing minerals, iron oxides, and other mineral phases, depending on the source material and processing route. Their particle sizes may range from coarse sand-sized fractions to very fine silt-like particles, while particle morphology can vary from angular to sub-angular owing to crushing, grinding, and beneficiation processes [19,20,21,22,24]. This broad variability distinguishes mining tailings from more standardised waste-derived materials such as FA and represents both an opportunity and a challenge for their utilisation in cementitious systems.

A major advantage of mining tailings is their widespread availability and potential to reduce the environmental burden associated with long-term tailings storage [5,6,7,8,34]. Their utilisation in cementitious materials can contribute to waste valorisation, resource conservation, and reduced dependence on natural aggregates and conventional raw materials [19,20,21,22,24]. Furthermore, certain tailings contain silica, alumina, or calcium-rich phases that may provide beneficial physical or cementitious characteristics depending on the source material and degree of processing [20,24]. The potential to utilise large volumes of material while simultaneously reducing mining waste inventories has made tailings an attractive candidate for sustainable construction applications [19,20,21,22,24].

Despite these advantages, several limitations restrict the widespread utilisation of mining tailings. The most significant challenge is the substantial variability in physical, chemical, and mineralogical properties between tailing sources [19,20,21,22,24]. Unlike FA, which is produced under relatively controlled industrial conditions and is governed by established standards [18], tailings often require extensive characterisation before incorporation into cementitious systems [19,20,21,22,24]. Differences in particle grading, mineral reactivity, moisture content, and storage history can significantly influence their suitability for construction applications [19,20,21,22,24].

In addition, some tailings may contain elevated concentrations of potentially harmful constituents or contaminants that require careful assessment and management prior to utilisation [6,7,8]. Depending on the source, additional processing such as classification, beneficiation, grinding, or chemical treatment may be required to achieve suitable material properties, increasing implementation costs and complexity [19,20,21,22,24]. Because tailing characteristics are highly site-specific, findings reported for one tailing source cannot necessarily be generalised to others, highlighting the importance of source-specific assessment and material characterisation [19,20,21,22,24].

Overall, mining tailings represent a highly diverse class of waste-derived materials with significant potential for utilisation in cementitious systems. However, their successful application depends on comprehensive material characterisation, source-specific evaluation, and appropriate processing to ensure consistent performance and environmental compatibility.

Although CGA, FA, and mining tailings are all considered waste-derived materials, their physical characteristics, chemical composition, and governing mechanisms differ substantially. These differences influence their behaviour in cementitious systems and ultimately govern their effects on fresh-state and hardened-state properties. Figure 1 summarises the principal characteristics and mechanisms associated with each material and their relationship to performance.

As illustrated in Figure 1, the performance of waste-derived materials is governed by distinct mechanisms. CGA primarily influences behaviour through particle packing, aggregate geometry, and interfacial transition zone characteristics, whereas FA contributes through pozzolanic reactions and secondary C–S–H formation. In contrast, mining tailings exhibit more variable behaviour owing to differences in mineralogical composition, particle characteristics, and source-dependent reactivity. These mechanisms collectively influence fresh-state and hardened-state performance and provide the basis for the comparative assessment presented in the following sections.

Table 1 and Figure 1 provide a comparative synthesis of the characteristics, mechanisms, and performance trends of waste-derived materials in cementitious systems. Representative studies are presented in Table 2, Table 3 and Table 4 to illustrate the principal findings discussed throughout the review. A complete summary of all reviewed studies is provided in Supplementary Tables S1–S3.

This table summarises six representative studies highlighting optimal replacement ranges, mechanical performance, workability trends, and durability implications. Full details of all 23 reviewed CGA studies are provided in Supplementary Table S1.

Table 3 presents five representative studies highlighting optimal replacement ranges, mechanical performance, workability trends, and durability implications. A comprehensive database of all reviewed FA studies is provided in Supplementary Table S2. While the macroscopic behaviour of FA in cementitious systems is relatively well established, understanding of the underlying hydration mechanisms and microstructural evolution remains limited.

A summary of representative studies is provided in Table 4, while a comprehensive database of all reviewed mining tailings studies is provided in Supplementary Table S3.

The representative studies presented in Table 2, Table 3 and Table 4 demonstrate that the effects of waste-derived materials on cementitious systems are governed by a combination of particle characteristics, chemical composition, replacement level, and mixture design. The following sections compare the influence of CGA, FA, and mining tailings on fresh-state behaviour, mechanical performance, durability, and the governing mechanisms responsible for these observed trends.

## 3. Comparative Evaluation

### 3.1. Crushed Glass Aggregate (CGA)

Across the 23 reviewed studies on CGA (Supplementary Table S1), clear trends emerge in relation to workability, mechanical performance, and durability. At low to moderate replacement levels, most studies report that CGA maintains or improves workability and compressive strength relative to natural sand [27,35,36,37]. In contrast, higher replacement levels (>50%) generally result in reduced strength and increased slump loss, which are commonly attributed to the combined effects of angular particle geometry, reduced packing density, and increased water demand [45,47].

Reported favourable replacement levels vary according to application and replacement strategy. Conventional concrete studies generally report favourable performance at low to moderate CGA replacement levels [23,27,40], whereas Jiao et al. [37] reported maximum compressive strength at 75% glass aggregate replacement in UHPC systems. Shotcrete studies identified favourable performance at approximately 15–20% replacement [51,52]. These findings indicate that favourable replacement levels are application-dependent and that no single optimum value can be generalised across all CGA applications. Some specialised or high-performance mixes demonstrate acceptable performance even at 50–75% replacement [26,48], although such outcomes are strongly dependent on particle size distribution, surface texture, and mix-design optimisation. These variations highlight the sensitivity of CGA-modified mixtures to grading, angularity, and chemical composition, all of which directly influence packing density and water demand.

Durability trends are comparatively consistent. Moderate CGA contents (approximately 15–25%) typically reduce water absorption and enhance resistance to chloride ingress [23,27]. Conversely, excessive glass contents increase susceptibility to freeze–thaw deterioration and may elevate the risk of alkali–silica reaction (ASR) in systems lacking appropriate mitigation strategies [49,50].

Although these mechanical and durability trends are well documented, only a limited subset of CGA studies investigates the underlying mechanisms governing performance, such as hydration behaviour, ITZ development, or pore structure evolution [38,50]. Most publications infer mechanisms indirectly from macroscopic test results rather than from direct microstructural evidence. Consequently, significant knowledge gaps remain regarding the influence of CGA on hydration kinetics, pore refinement, and ITZ morphology.

Shotcrete-specific studies demonstrate consistent trends in CGA performance. Basireddy et al. [51] and Serati et al. [52] both reported increased workability, pumpability, and bleeding with increasing CGA content, primarily due to the low water absorption and angularity of glass particles. Mechanical performance was generally maintained for up to 50% replacement, with optimal ranges identified between 15–20%. Strength reductions at higher replacement levels were attributed to coarse particle size distribution and reduced packing efficiency. Augonis et al. [53] further demonstrated that expanded glass aggregates can improve mechanical performance when combined with suitable admixtures, with strength gains linked to ITZ densification and improved particle packing.

The broader literature demonstrates widespread utilisation of waste glass across civil engineering applications, including conventional concrete [23,27,28,35,38,39,40,41,42,43,44,45,46], high-strength concrete [36], ultra-high-performance concrete (UHPC) [37], structural concrete [54], pervious concrete [25,55], self-compacting concrete [48,49], geotechnical fills [26], road materials [47], mortar [56], and shotcrete [51,52,53], highlighting its versatility as a sustainable construction material.

These findings demonstrate that CGA is a viable aggregate replacement at moderate substitution levels; however, performance remains strongly dependent on particle grading, morphology, and mix design. The following section therefore examines the available evidence on hydration kinetics, microstructural development, and ITZ behaviour in FA-modified cementitious systems.

#### Hydration Mechanisms and Microstructural Development of CGA Systems

The hydration and microstructural behaviour of cementitious systems incorporating CGA has been reported far less frequently than mechanical or durability performance, with only a limited number of studies providing direct microstructural evidence. In most CGA-related publications, microstructural behaviour is inferred from changes in strength, porosity, or durability, resulting in a notable gap in mechanism-level understanding.

Available microstructural studies indicate that the governing mechanisms depend strongly on the form in which recycled glass is incorporated. When recycled glass is processed into waste glass powder (WGP), pozzolanic reactions can influence hydration behaviour and pore structure evolution. Mercury intrusion porosimetry (MIP) analyses demonstrate that WGP progressively refines pore structure between 28 and 90 days due to reactions between dissolved amorphous silica and portlandite, resulting in secondary calcium–silicate–hydrate (C–S–H) formation [43]. This is evidenced by leftward shifts in pore-size distribution, reductions in capillary and macropores, increased gel pore fractions (<10 nm), and the development of a more tortuous and refined pore network at later ages.

Consistent observations have been reported in systems incorporating recycled glass sand, where increasing glass content reduces cumulative pore volume and overall porosity [38]. However, the relative contributions of physical effects (i.e., particle packing and porosity modification) and potential chemical interactions remain insufficiently understood, highlighting the need for further mechanistic investigation.

ITZ behaviour varies depending on replacement level and mix design. SEM analyses indicate that high CGA contents can lead to increased voids and microcracking within the ITZ, reflecting weakened particle bonding and reduced interfacial strength [48]. Conversely, in high-performance systems such as UHPC, improved particle packing and fluidity at moderate replacement levels can reduce ITZ cracking and enhance particle bonding [37]. These findings demonstrate that ITZ response to CGA is highly system-dependent and controlled by factors such as fluidity, particle grading, and surface texture.

Despite these findings, the majority of CGA studies do not include direct microstructural or hydration diagnostics such as SEM, MIP, X-ray diffraction (XRD), thermogravimetric analysis (TGA), or ITZ profiling, including several widely cited works [27,28,36,39,40,41,44,45]. These studies report macroscopic performance changes but do not directly characterise hydration reactions or microstructural development. Review-based analyses also discuss anticipated pozzolanic and microstructural effects but similarly lack primary experimental evidence and therefore cannot substitute for direct mechanistic investigation [23,25].

Overall, the limited mechanistic evidence suggests that CGA primarily influences performance through changes in pore structure and ITZ behaviour, while secondary C–S–H formation has been predominantly reported in studies incorporating finely ground waste glass powder (WGP). The scarcity of direct hydration and microstructural diagnostics represents a clear research gap, underscoring the need for mechanism-oriented studies in future CGA research.

### 3.2. Fly Ash (FA)

The reviewed studies on FA demonstrate consistent trends in workability, strength development, and durability when used as a SCM (Supplementary Table S2). At low replacement levels (≤15%), FA generally maintains structural performance in shotcrete and concrete systems [29,31,32]. Higher replacement levels (>30%) commonly result in reductions in early-age strength due to slower hydration kinetics [31,32], although improved long-term strength and durability performance are frequently observed at FA contents between 20% and 30%, particularly when ultrafine FA particles (<5 μm) are used to enhance pozzolanic activity [32].

Workability improvements are widely reported and are primarily attributed to the spherical morphology of FA particles, which reduces internal friction and water demand [29,32]. Durability benefits include reduced permeability and enhanced resistance to chloride ingress at moderate FA contents (approximately 15%) [33]. However, excessive FA contents may delay strength development, particularly under cold-climate curing conditions [29].

Variability in favourable substitution rates primarily arises from differences in particle fineness, calcium content, curing regime, and mix design. Although numerous studies assess mechanical performance, relatively few of the reviewed shotcrete studies investigate hydration mechanisms or microstructural evolution in detail, and long-term durability under aggressive environmental exposures remains insufficiently studied. These limitations highlight the need for standardised testing protocols, life-cycle assessments, and mechanistic investigations to validate the practical implementation of FA in shotcrete applications. While the macroscopic behaviour of FA-cement systems is relatively well established, understanding of the underlying hydration mechanisms and microstructural evolution remains limited for FA-modified shotcrete.

#### Hydration Mechanisms and Microstructural Development of FA Systems

The hydration and microstructural behaviour of cementitious systems incorporating FA are reported far less frequently in shotcrete-focused literature than mechanical and workability outcomes. A significant proportion of studies examining FA in shotcrete or conventional concrete [29,31,32,33] evaluate only fresh-state properties and strength development, without providing direct microstructural or hydration diagnostics such as SEM, MIP, XRD, TGA, or calorimetry. Consequently, direct mechanistic evidence within the reviewed FA-modified shotcrete studies remains limited, particularly regarding its influence on hydration kinetics and ITZ development.

Available mechanistic insights derived from broader SCM studies indicate that FA exhibits minimal reactivity at early ages, with its primary influence governed by dilution of the cement content [18]. At replacement levels exceeding approximately 10%, both peak hydration intensity and early cumulative heat release decrease, reflecting a reduced supply of reactive clinker phases. FA incorporation also increases initial setting time and does not significantly accelerate hydration within the first 20 h, confirming its limited contribution to early-age hydration processes [18].

Beyond early-age behaviour, the FA-based shotcrete and concrete studies included in this review provide limited direct microstructural evidence addressing:

- ITZ morphology,
- pore-size distribution or pore-refinement trends,
- calcium hydroxide (CH) consumption,
- secondary C–S–H formation, or
- long-term microstructural evolution.

Studies examining FA in shotcrete systems report macroscopic performance measures, including compressive, tensile, and flexural strength, as well as rebound behaviour and spray performance, but do not include direct hydration or microstructural diagnostics [29,31,32,33]. As a result, the influence of FA in shotcrete systems is primarily inferred as early-age dilution combined with delayed hydration effects, without direct experimental confirmation of ITZ modification, pore refinement, or later-age hydration products.

Within the reviewed shotcrete literature, microstructural and hydration-focused investigations are comparatively scarce, limiting the mechanistic evidence currently available for FA-modified shotcrete. Future studies should incorporate complementary techniques such as SEM, MIP, XRD, TGA, and calorimetry to establish a mechanistic understanding of FA behaviour in shotcrete systems and to validate assumptions commonly extrapolated from conventional FA–cement literature.

### 3.3. Mining Tailings

The 45 reviewed studies on mining tailings demonstrate highly variable performance outcomes depending on mineral origin, particle morphology, and chemical composition. Iron ore tailings (IOT) generally exhibit comparable or improved compressive strength when used as fine-aggregate replacements at levels of approximately 20–40% [70,75,80]. In some ultra-high-performance concrete (UHPC) systems, optimal mechanical performance has been achieved through combined substitutions of IOT powder and sand, indicating a favourable balance between packing density and binder interaction at moderate replacement levels [71]. Gold tailings show promising durability performance, particularly enhanced resistance to chloride ingress and freeze–thaw cycling at replacement levels of 25–30% [82,83]. However, reductions in workability are frequently observed due to the angular particle shape and increased surface roughness of gold tailings. Copper tailings, by contrast, tend to improve workability owing to their higher density and smoother particle texture, but reported strength performance becomes inconsistent at replacement levels exceeding 40%, with some studies indicating strength reductions [86,100].

Bauxite tailings (commonly referred to as red mud) exhibit the greatest variability in reported performance. When used as partial cement replacements at relatively low levels (10–20%), bauxite tailings have been shown to enhance compressive strength and durability through pozzolanic and filler effects [24,62]. At higher replacement levels, however, significant reductions in workability and early-age strength are commonly reported [60,67]. Microstructural investigations attribute these trends to a combination of physical filler effects, densification of the ITZ, and the formation of secondary hydration products such as C–S–H and ettringite, particularly in thermally activated bauxite tailings systems [65,68].

Despite reported strength and durability benefits at moderate substitution levels, data on long-term durability remains limited and inconsistent. Relatively few studies address sulfate resistance, carbonation, or ASR mitigation in tailings-based systems, particularly for shotcrete applications. Consequently, the lack of standardised testing protocols and life-cycle assessment frameworks represents a significant barrier to the practical adoption of mining tailings in shotcrete and concrete. Establishing consistent methodologies for materials characterisation, performance evaluation, and environmental assessment is therefore essential to support wider field implementation.

#### 3.3.1. Applications, Material Variability and Literature Overview

Mining tailings have been widely investigated as replacements for aggregates and SCMs, with performance governed by mineral origin, composition, particle morphology, and processing conditions [20,21,22,24,62]. The reviewed literature encompasses a wide range of tailing types, including iron ore, gold, copper, coal, and bauxite tailings, as well as mixed mineral systems and FA-based combinations [18,19,24,29,33,57,59,60,61,62,63,64,65,66,67,68,69,70,71,72,73,74,75,76,77,78,79,80,81,82,83,84,85,86,87,88,89,90,91,92,93,94,95,100,101].

The utilisation of mining tailings offers sustainability benefits linked to their chemical and mineralogical composition, waste-reduction potential, and availability in large volumes. However, the reviewed literature reports conflicting results regarding favourable replacement rates, reflecting the significant variability in tailings composition and physical characteristics.

Focusing specifically on shotcrete applications, published studies include investigations involving iron ore tailings [59,72] (and Liu et al., cited in [72]), gold tailings [81] (and Lilies et al., cited in [82]), coal tailings [89,90], FA-based systems [29,33] (including Wu et al. and Lu et al., cited in [29]), and rheological studies incorporating tailings [102]. Notably, no published literature was identified on the use of bauxite tailings in shotcrete, highlighting a significant research gap. A comprehensive summary of all reviewed mining tailings studies is provided in Supplementary Table S3.

The literature indicates that different types of tailings influence concrete and shotcrete performance in distinct ways. When tailings are used as fine aggregates, workability generally decreases, whereas compressive strength often increases. Conversely, when used as SCMs, strength may decrease at higher replacement levels due to reduced clinker content and limited pozzolanic activity in some tailings. Despite variability in reported replacement levels and performance trends, there is broad agreement that mining tailings can be feasibly incorporated into cementitious systems.

Iron ore tailings typically possess a rough surface texture and high specific surface area, which can locally reduce the effective water-to-cement ratio and enhance bonding with the cement paste, contributing to strength development [80]. Gold tailings often exhibit finer particle sizes and measurable pozzolanic activity, leading to improvements in compressive and tensile strength [82]. Copper tailings, characterised by high specific gravity, tend to increase concrete density, compressive strength, and abrasion resistance while reducing chloride permeability and drying shrinkage [86]. Coal tailings generally show limited susceptibility to ASR and exhibit physical and mechanical behaviour comparable to conventional aggregates [103]. Bauxite tailings have been reported to improve mechanical strength, reduce chloride penetration, and accelerate pozzolanic reactions due to their elevated iron oxide and aluminium oxide contents [61].

Comparative reviews of iron, copper, and gold mine tailings highlight the influence of mineralogy and particle characteristics on performance. Aggregate-based systems typically exhibit reduced workability while maintaining acceptable compressive strength at moderate replacement levels, although copper tailings may improve workability and show greater variability in strength performance at higher contents [22]. Gou et al. [20] reported that aggregate replacement levels above approximately 30% were frequently associated with reduced performance in several tailings systems; however, the same review emphasised the substantial variability in tailings mineralogy, chemistry, and physical characteristics. Consequently, replacement levels should be considered tailing-specific and application-specific rather than universally applicable. When tailings are used as cement replacements, performance is governed by the balance between dilution effects and available pozzolanic reactivity. In many cases, limited pozzolanic activity results in reduced compressive strength at higher substitution levels, while moderate replacement levels may provide beneficial filler and secondary hydration effects. However, the extent of these benefits is strongly dependent on mineralogical composition and processing history [20].

A key finding across the literature is the significant variability in durability performance. While some studies report improvements in chloride resistance and reduced permeability, results for sulfate resistance, carbonation, and long-term durability remain inconsistent. This variability is closely linked to differences in chemical composition, as illustrated in Table 5, which highlights the wide range of oxide contents across tailing types.

Recent reviews also emphasise the need for improved standardisation in testing and evaluation methods. The absence of consistent durability data and the lack of unified characterisation approaches limit the ability to directly compare results across studies and hinder large-scale implementation. In addition, system-level considerations, including density control and overall mix design optimisation, have been identified as critical factors influencing practical performance, particularly for high-density tailings systems [21].

In shotcrete applications, performance is further influenced by mix design parameters, including water-to-binder ratio, admixture dosage, and fibre content. These factors govern fresh-state behaviour and structural integrity, often outweighing the direct effects of tailings substitution alone. Consequently, optimisation of mix design is essential to achieve reliable performance in sprayed systems [33].

Overall, mining tailings exhibit highly material-dependent behaviour. Favourable replacement levels vary substantially according to tailing type, mineralogy, particle morphology, processing history, and functional role within the cementitious system. Iron ore, gold, copper, coal, and bauxite tailings exhibit distinct mechanical, durability, hydration, and microstructural behaviours; therefore, no single favourable replacement level can be generalised across all tailing systems. Performance is primarily governed by particle morphology, mineralogy, and processing conditions, with beneficial outcomes observed when packing density, filler effects, and secondary hydration mechanisms are appropriately utilised. Despite these advantages, variability in composition and the limited availability of long-term durability data present significant challenges to standardisation and large-scale implementation. These findings provide a basis for comparison with other waste-derived materials, particularly when considered within the framework of their distinct functional roles in cementitious systems.

#### 3.3.2. Hydration Mechanisms and Microstructural Development of Mining Tailings Systems

##### Iron Tailing

The hydration and microstructural behaviour of cementitious systems incorporating iron-ore tailings (IOT), including iron-ore tailing powder (ITP) and iron-ore tailing sand (ITS), depends strongly on mineralogy, fineness, activation state, and replacement level. Compared with the mechanical and durability data available in the literature, fewer studies provide direct hydration or microstructural diagnostics. However, where such evidence is available, three dominant mechanisms are observed: (i) physical filling and packing effects, (ii) nucleation-driven acceleration or modification of hydration, and (iii) conditional pozzolanic activity, particularly in siliceous or mechanochemically activated tailings.

Several studies highlight the importance of filler and packing effects, particularly at moderate replacement levels. In wet-mix shotcrete containing waste rock and tailings sand, SEM analyses show that increasing cement content or mass concentration reduces internal voids and increases C–S–H infill between structural planes, producing a denser cementitious skeleton and significant strength gains [59]. Similar effects are observed in magnesium oxysulfate cements (MOS), where IOT reduces macropores and refines pore structure through physical filling, although phase analysis confirms that IOT does not actively participate in hydration reactions within the pH range of MOS systems [72]. In UHPC, moderate IOT sand contents (≤60%) refine pore structure and reduce porosity, with MIP and water absorption tests demonstrating densification of the matrix due to fine-particle filling and reduced harmful (>100 nm) pores [70].

Nucleation effects are also observed in cement pastes and UHPC mixes containing ITP. Isothermal calorimetry consistently shows that IOT/ITP reduces the magnitude of the C3S hydration peak (dilution) while sharpening and advancing the aluminate hydration peak. This behaviour indicates that finely ground IOT particles act as nucleation sites, accelerating aluminate reactions and modifying early hydration kinetics without necessarily consuming CH [78]. In MOS systems, HEDP alters hydration pathways and promotes the formation of the 5·1·7 phase (5Mg(OH)_2_·MgSO_4_·7H_2_O), but IOT continues to behave as an inert filler rather than a reactive phase [72].

Whether iron tailings exhibit pozzolanic activity depends strongly on composition and activation. Some studies conclude that ITP lacks pozzolanic activity, with chemical activity curves, TGA, and MIP all confirming an absence of CH consumption and behaviour dominated by microaggregate filling [74,78]. However, other work demonstrates that siliceous and mechanochemically activated IOT can exhibit secondary hydration. In concrete with 20–30% activated siliceous tailings, TGA indicates reduced CH contents at 28 days, SEM shows more compact flocculent C–S–H, and XRD confirms reduced portlandite peak intensity. Together, these findings show that activated tailings can participate in secondary C–S–H formation, partially compensating for reduced clinker content and densifying the hardened microstructure [79]. Similarly, in IOT–cement blends, siliceous tailings reduce portlandite content relative to the reference, suggesting a partial pozzolanic reaction [76], although this behaviour is not universal across all tailings types.

ITZ behaviour varies considerably with tailings type and replacement level. SEM analyses of UHPC containing IOT sand show that moderate replacement levels (20–60%) produce a dense, well-bonded ITZ with a homogeneous C–S–H distribution, correlating with improved mechanical properties and reduced porosity [70]. At very high replacement levels (100% IOT), SEM reveals entrapped air voids, AFt accumulation, and microcracking around the ITZ, leading to degraded strength and stiffness [70,71]. In systems containing ITP or ITS, ITZ quality is sensitive to particle size distribution. Smaller IOT particles improve ITZ bonding by increasing specific surface area, enhancing dispersion within the paste, and reducing localized stress concentrations, while coarser or irregular particles promote ITZ cracking and stress amplification during loading [80].

Cyclic loading studies provide additional insights into microstructural evolution. Discrete element method simulations calibrated against laboratory tests reveal that fractures tend to initiate around the IOT skeleton, where stress concentrations develop under cyclic loading. Higher IOT contents form a more stable internal skeleton, improving resistance to cyclic damage and reducing strain-softening compared to mixes with lower IOT contents [80]. Particle size also governs crack propagation, with smaller IOT particles reducing fracture density by improving the uniformity of paste–aggregate bonding and limiting the development of large ITZ flaws.

Overall, the hydration and microstructural behaviour of iron-tailings systems is governed by a balance between filling, nucleation, and conditional pozzolanic activity. Moderate replacement levels consistently refine pore structure, densify the ITZ, and improve impermeability, whereas very high replacement levels may introduce voids, AFt accumulation, or microcracking that degrade mechanical properties. Pozzolanic reactions occur only under specific conditions, typically when tailings are siliceous and/or mechanochemically activated, while most tailings act primarily as microaggregate fillers. The limited number of studies pairing SEM, MIP, XRD, TGA, and calorimetry highlights a substantial research gap and reinforces the need for more mechanism-oriented investigation into tailings-modified cementitious systems.

##### Gold Tailings in Shotcrete

Due to the available gold tailings literature, including both shotcrete-specific systems and conventional concrete/UHPC systems with distinct hydration and microstructural behaviours, these mechanisms are discussed separately for clarity. In shotcrete systems, SEM–EDS analyses show that graded gold tailings wet shotcrete develops a typical assemblage of cement hydration products, including amorphous C–S–H gel, spherical ettringite (AFt), plate-like calcium hydroxide (CH), gypsum, mullite, and quartz [81]. Over curing from 28 to 56 days, large CH plates and gypsum crystals progressively transform into a denser network of fibrous and reticular C–S–H, accompanied by increased AFt content. This evolution corresponds with increasing compressive strength and reflects progressive microstructural densification. The incorporation of graded tailings together with supplementary fines (i.e., silica fume, slag, and FA) further promotes pore filling and homogenisation of hydration products, enhancing matrix compactness [81].

Additional microstructural evidence indicates that particle gradation strongly influences paste–aggregate interaction. Systems with high tailings content (i.e., 100%) exhibit reduced paste film thickness due to increased paste demand from finer particles, whereas blended systems (i.e., ~50% replacement) achieve improved packing through combined coarse and fine fractions [83]. This results in a more uniform distribution of hydration products and improved ITZ quality. Excessive tailings contents, however, may reduce stress transfer efficiency due to insufficient paste coverage.

Taken together, hydration in gold tailings shotcrete is characterised by progressive C–S–H and AFt formation, a reduction in CH and gypsum, and improved densification with appropriate gradation and fines content. Particle packing, microstructural homogenisation, and ITZ refinement are the dominant mechanisms governing performance at moderate replacement levels.

##### Gold Tailings in Concrete/UHPC

In conventional concrete, microstructural analyses using SEM, XRD, EDS, and MIP demonstrate that gold tailings significantly influence matrix densification and pore structure [82]. At the replacement level reported to produce the most favourable performance (30%), dense microstructures with high C–S–H content and minimal microcracking are observed, whereas lower or higher replacement levels exhibit increased porosity or re-emergence of microcracks. Reduced Ca/Si ratios and substantial porosity reductions (up to 45%) are consistent with increased secondary C–S–H gel formation, which corresponds directly to improved durability performance.

In UHPC systems, the curing regime is a critical factor governing hydration behaviour [84]. Hygrothermal curing enhances hydration and C–S–H formation, while combined thermal regimes promote the development of crystalline hydrates such as tobermorite (Ca/Si ≈ 1.01), associated with improved mechanical performance. These effects are attributed to autoclave-like conditions, where elevated temperature and internal vapour pressure facilitate continued hydration and crystallisation.

Overall, the concrete and UHPC studies show that gold tailings influence hydration and microstructure primarily through particle-packing and filler effects, which densify the matrix and reduce porosity at favourable replacement levels. Latent silica and alumina in the tailings can contribute to additional C–S–H formation under favourable curing conditions, particularly in UHPC. However, excessive tailings content (i.e., ≥40%) disrupts optimal packing, introduces microporosity, and diminishes microstructural integrity. Combined with the shotcrete findings, these results highlight the importance of carefully balancing tailings content, particle size distribution, and curing regime to maximise the beneficial hydration and microstructural outcomes of gold-tailings incorporation.

##### Bauxite/Red Mud (RM)

*RM as OPC Replacement (cementitious binder role)*

Across Portland-based systems, RM participates in hydration and microstructure development through a coupled filler–nucleation effect at early ages and conditional pozzolanic/hydraulic participation at later ages or after activation. SEM observations of RM pastes show that, from 7 → 28 → 90 days, there is a progressive reticulated C–S–H coating of unhydrated grains, with needle-like AFt and crystalline CH evident early and a denser gel network later; EDS indicates decreasing Ca with increasing Si and Al in the hydrates over time, consistent with continued gel formation and matrix consolidation [24]. ITZ imaging within concrete confirms that high RM contents can yield a more porous ITZ with microcracking when RM’s intrinsic porosity and water uptake dominate; conversely, low–moderate dosages (and/or fine RM) tighten the ITZ as gels and fines infill the interfacial voids [24].

Calorimetry and paste XRD across several studies indicate that RM raises the aluminate availability in the pore solution, often intensifying early AFt formation and modifying induction; later-age signatures show reduced CH peaks where RM participates chemically, while C–S–H/AFm/AFt remain the dominant hydrates [94,97,98,99]. In clinker-free binders (pastes/mortars), TGA mass-loss windows associated with C–S–H/AFt (≈60–200 °C) and CH (≈430–520 °C) confirm relative hydrate content; where RM is sufficiently fine and reactive, CH residue decreases relative to reference pastes at equal age, evidencing CH consumption and additional hydrate formation [97].

The pozzolanic nature of RM is composition- and treatment-dependent. Salim et al. [24], standard solution tests (NP EN 196–5) show rapid CH consumption from the pore solution when RM is blended with cement, confirming pozzolanicity in an aqueous cementitious environment. However, without pretreatment, some datasets report weak or inconsistent pozzolanic activity, emphasising that fineness, alkalinity, and mineralogy govern whether RM acts primarily as a microfiller/nucleant or also as a reactive aluminosilicate [24].

Overall, for OPC systems, the microstructural signature is:

i. early densification via filler/nucleation,
ii. time-dependent CH consumption and C–(A)–S–H growth where chemistry permits,
iii. an ITZ that densifies at low to moderate RM contents but becomes porous at high contents due to RM’s water demand and intrinsic porosity [24].

*RM as Fine Aggregate/Sand (3D-printed mortar matrix)*

When bauxite tailings replace natural sand, microstructure improvements arise primarily from particle packing and nucleation rather than bulk binder chemistry, with a clear optimum near 35% in the investigated 3D-printing mortar. Isothermal calorimetry shows that the induction period ends earlier and the second heat-release peak occurs sooner when 35% tailings are used, indicating an abundant nucleation substrate for hydrate precipitation [93]. MIP demonstrates that the addition of tailings shifts pore sizes to smaller diameters and reduces cumulative pore volume relative to the no-tailings reference; the fraction of “more detrimental” (>200 nm) pores becomes minimal at the optimum replacement, directly evidencing pore-network refinement [93].

Nanoindentation focused on the ITZ finds markedly lower porosity and increased fractions of HD/UHD C–S–H and CH at the optimum, reflecting a denser interfacial skeleton rather than a chemically altered hydrate assemblage [93]. TGA at equal age shows a larger total hydrate mass (C–S–H/AFt window) for the optimum tailings mix than for the reference, while CH mass is also higher, implying that net CH generation from cement hydration exceeds CH consumed by any limited tailings reactivity in this matrix [93]. SEM corroborates a tight paste–tailings interface with hydrate coatings on tailings surfaces, consistent with filling + nucleation; at excessive replacement (i.e., 45%), extrudability-induced voids and interlayer defects re-introduce porosity and disrupt the refined pore structure [93]. In short, for RM as sand, the mechanism is physical densification of the pore system and ITZ via filler/nucleant effects, with only minor chemical participation at the binder scale.

*Clinker-Level Interaction (RM in raw mix and clinker hydration)*

At the kiln scale, using RM in the raw mix does not change the clinker phase types; C_3_S/C_2_S/C_4_AF remain dominant, but it does shift proportions, notably increasing C_4_AF due to the iron content of RM [94]. SEM of clinkers shows larger alite crystals where RM raises the liquid phase and supplies mineralisation seeds (diaspore/Al-silicates), facilitating crystal growth [94]. During hydration of the resultant cements, XRD identifies typical hydrates C–S–H, AFt, and CH, while the CH peak in RM-bearing pastes becomes weaker at later ages than in the reference, consistent with a higher overall hydration degree or secondary consumption of CH within the blended system [94]. Thus, at clinker level, RM acts mainly as a process mineraliser and phase-balance modifier (↑C_4_AF), with downstream hydration reflecting a conventional OPC hydrate assemblage but subtly altered kinetics/quantities [94].

*Alkali-Activated/Geopolymer Systems (calcined tailings with slag)*

In alkali-activated matrices using calcined tailings with slag, long-term evidence shows a progressive reaction that reshapes pore structure and gel chemistry over years. XRD of pastes cured for up to 6 years reveals persistent inert minerals from the calcined tailings, but the emergence of carbonate products, calcite (from the carbonation of Ca-rich gels) and natron (from Na-rich pore solutions), indicates ongoing interaction between the gel network and ambient CO_2_ [95]. TG–DSC quantifies increasing calcite decomposition at approximately 700 °C, verifying progressive carbonation, while the endotherm near 120 °C shifts toward tighter water binding in matured gels, consistent with higher polymerisation and smaller gel pores [95]. MIP shows the disappearance of macropores present at 28 days, a reduced cumulative intrusion, and a pore-size distribution centred near 10 nm, reflecting gel infill and matrix densification, SEM images of aged pastes display fewer micrometer-scale voids and a more continuous gel texture [95]. Mechanistically, these binders show evidence of coexistence of multiple gel types; C–A–S–H dominates at early ages due to slag hydration, while N–A–S–H becomes increasingly prominent at later ages as aluminosilicate geopolymerisation progresses. The resulting microstructural network is a stable, cross-linked gel whose evolution continues well beyond conventional cement curing windows [95].

*Thermal Activation and Reactivity Tuning*

Thermally activating bauxite tailings/red mud increases reactivity by transforming key minerals into alumina-bearing minerals. XRD, FTIR, and TGA analyses show that diaspore dehydroxylates to corundum at approximately 600 °C, while kaolinite fully transforms to metakaolin near 700 °C, maximising Si/Al availability and surface activity before sintering and recrystallisation begin to reduce reactive sites [65]. In blended pastes, TGA then demonstrates minimum CH residue at 700 °C activation, i.e., maximum CH consumption and thus maximum secondary hydrate formation at equal age [65]. Activity indices likewise peak at 700 °C, especially for the finer classified fraction, confirming that fineness + 700 °C calcination is an effective route to elevate pozzolanic participation [65]. In OPC systems, this shifts RM behaviour from predominantly filler/nucleant to reactive aluminosilicate capable of adding C–(A)–S–H/C–A–S–H, while in alkali-activated systems it accelerates aluminosilicate dissolution and network development.

*Mechanistic Synthesis (RM)*

1. OPC replacement: Early-age filler/nucleation dominates; later-age CH consumption and C–(A)–S–H growth occur where fineness/chemistry permit. ITZ densifies at modest RM; high RM can increase ITZ porosity due to particle porosity and water demand [24].
2. Sand replacement (3DPC): Microstructure improves via packing and nucleation, MIP-verified reduction of harmful pores and nanoindentation-verified ITZ densification at 35%; chemical participation is limited relative to OPC replacement [93].
3. Clinker level: RM increases C_4_AF, acts as a mineraliser/seed, and yields conventional hydrates with subtle CH evolution indicative of altered kinetics/degree [94].
4. Alkali-activated: Long-term progressive geopolymerisation leads to pore refinement, carbonate formation (calcite, natron), and a gel balance trending to N–A–S–H at later ages, with stable, tightly bound water signatures [95].
5. Activation: ~700 °C is an effective optimum for converting diaspore/kaolinite to corundum/metakaolin, maximising Si/Al release, CH consumption, and activity index; excessive temperatures promote sintering and reduce reactivity [65].

##### Coal Gangue/Coal Tailings

Coal gangue (CG) is characterised by a mineral assemblage dominated by kaolinite, illite, and quartz, with minor chlorite/smectite and carbonate/sulfide phases, resulting in fines with a high specific surface area, high water-uptake capacity, and platy clay fabrics. These properties strongly influence fresh-state behaviour and, by extension, the development of the ITZ. In shotcrete systems, limited hardened-state microstructural diagnostics are available; however, mineralogical characteristics and fresh-state behaviour allow for mechanistic inference. Clay-rich, angular gangue fines withdraw mixing water and disrupt lubrication, reducing slump unless partially offset by rounded aggregates such as river sand. This increased water demand, combined with platy clay morphology, is likely to increase early ITZ porosity unless sufficient paste and improved particle grading are present. Conversely, mixtures incorporating appropriate proportions of rounded aggregates maintain workable rheology and improved packing, resulting in a more compact ITZ structure [90].

SEM analyses indicate that particle size and surface morphology of coal gangue govern ITZ formation [89]. Finer (~100 µm) polygonal particles are tightly encapsulated by hydration products, producing dense matrices with minimal interfacial cracking. In contrast, coarser particles (200–500 µm) exhibit increased pore formation and microcracking within the ITZ, indicating weaker mechanical interlock. Internal defects within gangue particles can redistribute water and act as internal curing reservoirs, with their influence dependent on the balance between defect density and hydration progression. Fibre incorporation further enhances interfacial integrity by bridging microdefects and limiting crack propagation [89].

Multiscale analyses incorporating SEM, EDS, and nuclear magnetic resonance (NMR) demonstrate that supplementary cementitious materials, particularly FA, significantly modify hydration and microstructural development [91]. At optimal FA contents (~30%), the ITZ becomes denser with reduced porosity and limited microcracking, as FA particles act as nucleation sites and micro-aggregates that refine the pore structure. Secondary hydration processes promote the formation of additional C–S–H through CH consumption, while AFt/AFm formation contributes to further densification. NMR results confirm pore refinement through reduced macropore volume and increased gel pore fractions. At suboptimal FA contents (i.e., 10% or 40%), insufficient CH consumption or paste deficiency results in increased pore connectivity and re-emergence of ITZ cracking [91].

Taken together, these findings demonstrate that hydration and microstructural development in coal gangue systems are highly sensitive to particle size, mineralogy, and supplementary cementitious material content. Where microstructural diagnostics are available [89,91], CG systems exhibit a wide range of ITZ behaviours, from dense and well-bonded to porous and microcracked, depending on particle characteristics and mix design. In the absence of detailed diagnostics, mechanistic understanding relies on mineralogical and fresh-state behaviour, highlighting the need for integrated rheology–microstructure–hydration studies in shotcrete systems. Overall, CG influences hydration primarily through its control of local water distribution, ITZ densification, and secondary hydration processes, with FA acting as a key modifier of these mechanisms depending on dosage.

##### Copper Tailings

A review of the available literature on copper tailings and copper slag identified no studies providing microstructural diagnostics such as SEM, XRD, TGA, MIP, NMR, or ITZ characterisation. Consequently, no hydration-based or microstructure-supported mechanistic conclusions can be drawn for copper tailings at this stage. Reported effects in the literature [86,100] (i.e., changes in density, strength, permeability, or fresh-state behaviour) therefore cannot be linked to mechanistic pathways within the cementitious matrix without supporting microstructural evidence.

### 3.4. Combined Effects of Multiple Waste Materials in Cementitious Systems

Although most published studies examine waste materials in isolation, several investigations demonstrate that combining two waste-derived materials can produce beneficial combined effects through complementary physical and chemical mechanisms. These multi-material systems, spanning combinations of CGA, tailings, FA, RM, copper slag, waste foundry sand (WFS), and recycled aggregates (RA), highlight an emerging but underexplored pathway for enhancing hydration behaviour, microstructural refinement, and mechanical performance.

In self-compacting concrete (SCC) systems, combined use of CGA and recycled coarse aggregate (RCA) can offset the mechanical limitations typically associated with RCA. The smooth, low-absorption glass particles compensate for the high porosity and water demand of RCA, improving workability and promoting a more uniform paste distribution around aggregates. This results in reduced ITZ weaknesses and improved mechanical performance at optimised proportions [49].

Similarly, combinations of waste glass powder and plastic waste demonstrate complementary behaviour, where glass contributes silica-rich filler and pozzolanic effects, while plastic enhances deformability [42]. Although plastic alone reduces strength, combined systems can achieve balanced performance when sufficient glass content promotes matrix densification.

Other multi-material systems highlight the interaction between chemically active SCMs and physically active aggregates. Systems incorporating FA (30%) and WFS (10–30%) improve both workability and strength through simultaneous mechanisms, with FA enhancing secondary C–S–H formation and WFS improving packing density and reducing harmful porosity [58]. Comparable behaviour is observed in coal gangue–FA systems, where FA-generated hydration products fill microcracks around porous aggregates while angular gangue particles contribute mechanical interlock, resulting in a densified ITZ [91].

In mining waste systems, combined use of FA with iron tailings aggregates or tailings powder enhances early hydration kinetics through filler–nucleation effects, while FA provides additional pozzolanic contributions at later ages [73,77]. These systems also demonstrate improved durability performance, including reduced shrinkage, enhanced carbonation resistance, and lower chloride permeability, associated with refined pore structure and microstructural densification.

In metallic waste systems, combinations of FA and copper slag (CS) produce stable fresh-state behaviour and balanced mechanical properties, with FA contributing rheological stability and CS enhancing density and packing [100]. In alkali-activated systems, blends of slag and calcined tailings form hybrid C–A–S–H/N–A–S–H gel networks that evolve over extended curing periods, producing enhanced microstructural stability and pore refinement compared with single-material systems [95]. Similarly, FA and RM combinations promote additional aluminosilicate reactivity, improving strength development relative to individual components [66].

Across all studies, the observed combined effects can be grouped into three dominant mechanistic categories:

1. Filler–Nucleation Complementarity:
  Fine particles (i.e., tailing powders, glass fines) act as nucleation sites, while reactive SCMs (FA, RM, slag) provide aluminosilicate phases, collectively accelerating hydration and refining pore structure.
2. Mechanical and Packing Compatibility:
  Combining angular or porous aggregates (i.e., RCA, CGA, tailings) with smoother or finer materials (i.e., CGA, WFS, CS) improves gradation, reduces void content, and enhances ITZ integrity.
3. Chemical Complementarity:
  Pairing pozzolanic materials (FA, RM, WFS) with inert or semi-reactive components (glass, tailings, CS) enables simultaneous CH consumption, C–S–H formation, and matrix densification beyond what is achievable with single materials.

Despite these promising outcomes, the evidence base remains limited, with relatively few studies providing detailed microstructural diagnostics or systematically investigating ternary or quaternary systems. Nonetheless, the available results indicate that integrating multiple waste streams can produce distinct and complementary improvements in hydration behaviour and microstructural development, highlighting a key area for future research.

### 3.5. Practical Applicability and Implementation in Mining Operations

Shotcrete is consumed at extremely high volumes in underground mining and tunnelling, making material selection a major economic factor. Published industry data cited by Serati et al. [52] indicate that Australia used approximately 800,000 m^3^ of shotcrete in 2018, of which 500,000 m^3^ was used in underground construction.

The reviewed literature demonstrates that waste-derived materials, including CGA, FA, and mining tailings, can be incorporated into cementitious systems while maintaining acceptable engineering performance when used at appropriate replacement levels. These materials offer opportunities to reduce dependence on conventional construction materials while promoting resource efficiency and waste utilisation [18,20,24]. The use of CGA specifically supports landfill diversion, conservation of natural sand resources, and circular economy objectives through the beneficial reuse of waste glass in construction applications [1].

Among the reviewed materials, the strongest evidence for direct implementation in shotcrete is available for CGA [51,52]. Experimental studies demonstrate that CGA-based shotcrete can maintain or improve pumpability and workability while reducing water and binder demand. These characteristics may contribute to reduced material consumption and improved resource utilisation. However, detailed economic and life-cycle assessments were beyond the scope of the reviewed studies. Furthermore, no significant impairment of mechanical performance has been reported with sand replacement by crushed waste glass, highlighting its practical potential for shotcrete applications [52].

FA provides additional opportunities to reduce Portland cement consumption and associated carbon emissions through its supplementary cementitious behaviour [18,29,30,31,32,33], while mining tailings offer opportunities for resource recovery and the beneficial utilisation of materials that would otherwise require long-term storage and management [5,20]. Although implementation evidence for FA and tailings in shotcrete remains more limited than for CGA, the available studies indicate considerable potential for their incorporation into sustainable cementitious materials.

From a life-cycle perspective, replacing conventional aggregates and cementitious constituents with waste-derived materials can reduce landfill disposal and promote resource recovery [1], while decreasing demand for virgin materials and mitigating the environmental impacts associated with natural resource extraction [17]. As the mining and tunnelling sectors face increasing sustainability requirements and regulatory pressures, the reviewed studies indicate the technical feasibility of incorporating waste-derived materials into shotcrete and concrete systems. However, additional economic analyses and life-cycle assessments are required to quantify potential environmental and economic benefits.

#### Implementation in Existing Mining Projects and Barriers to Adoption

Available evidence indicates that CGA-based shotcrete can be produced using conventional shotcrete materials, admixtures, and wet-mix production procedures, suggesting compatibility with existing shotcrete manufacturing practices. Experimental studies have demonstrated that mixtures containing up to 50% sand replacement can satisfy key fresh and hardened performance requirements, including workability, pumpability, and mechanical strength [52].

Although FA and mining tailings have demonstrated potential as sustainable cementitious materials, evidence regarding their implementation in shotcrete applications remains limited compared with CGA. Consequently, the current understanding of practical deployment in underground shotcrete systems is largely based on studies involving CGA [51,52].

However, several literature-supported barriers to adoption remain. At higher replacement levels (i.e., 100%), reduced strength has been observed due to coarse particle size distribution, highlighting the importance of aggregate grading and quality control in material production. In addition, further investigation of long-term durability is required, including assessment of alkali–silica reaction (ASR) potential, thermo-mechanical behaviour, and performance under biaxial and triaxial stress conditions representative of underground environments [52].

More broadly, implementation challenges include ensuring consistent material quality, establishing reliable supply chains, aligning project specifications with the use of alternative aggregates, and validating performance under the variable environmental and geomechanical conditions encountered in mining operations. Addressing these challenges will be important for facilitating the large-scale adoption of waste-derived materials in underground shotcrete applications.

## 4. Identified Research Gaps

Several review studies have examined the utilisation of waste-derived materials in cementitious systems, including CGA, FA, RM, and mining tailings. Existing reviews have primarily focused on individual waste streams and their application in conventional concrete and mortar systems, with emphasis on mechanical performance, durability, and sustainability outcomes. While these studies provide valuable insights into material-specific behaviour, they rarely evaluate multiple waste materials within a unified framework or consider their comparative suitability for shotcrete applications.

Furthermore, most available reviews treat CGA, FA, and mining tailings as separate research domains, resulting in a limited understanding of their relative advantages, limitations, and functional roles within cementitious systems. Direct comparisons of these materials based on fresh properties, mechanical performance, durability characteristics, microstructural mechanisms, and practical implementation considerations remain scarce. This limitation is particularly evident for shotcrete applications, where engineering requirements differ substantially from conventional cast concrete due to the importance of rheology, pumpability, sprayability, rebound resistance, and early-age strength development.

Although individual studies have demonstrated the potential of CGA, FA, and mining tailings to improve sustainability and reduce reliance on virgin resources, a comprehensive assessment that integrates these materials within a common framework remains lacking. Evidence relating specifically to shotcrete is especially fragmented, with available studies predominantly focused on CGA, while investigations involving FA and mining tailings in shotcrete systems remain limited.

Therefore, this review provides an integrated synthesis of published studies examining the utilisation of CGA, FA, and mining tailings in cementitious materials. Unlike previous reviews that primarily assess individual waste streams, this study compares these materials within a common framework, evaluates their engineering performance, durability behaviour, and underlying mechanisms, and explores their implications for sustainable shotcrete development. Based on this synthesis, the following priority research gaps are identified.

Key Research Gaps:

1. Single-material bias and limited studies on combined waste-material systems: Most studies evaluate one waste stream at a time. There is minimal work on hybrid systems, particularly CGA–FA combinations, and no published shotcrete studies on FBMT either alone or blended with FA. The lack of studies evaluating the combined effects of waste-derived materials constrains understanding of how glass angularity and the spherical morphology of FA collectively influence packing density, water demand, rheological behaviour, and strength development. Furthermore, the absence of FBMT and FBMT-FA shotcrete studies restricts understanding of their fresh-state, mechanical, and durability performance.
2. Rheology and pumpability remain under-characterised: While many studies report slump or flow values, true rheological characterisation remains limited for mixes incorporating hybrid systems. Pumpability windows, hose pressure losses, sprayability behaviour, rebound characteristics, and nozzle-exit velocity profiles have not been systematically evaluated against variables such as water-to-cement ratio, superplasticiser dosage, and particle size distribution. This gap is particularly significant for shotcrete applications, where fresh-state performance strongly influences placement quality and operational efficiency.
3. Durability under aggressive exposures is insufficiently resolved: Evidence relating to chloride ingress, sulfate resistance, carbonation depth, freeze–thaw performance, and ASR mitigation remains fragmented, particularly for blended waste systems. Long-term durability data extending beyond one to two years is scarce, while studies investigating coupled exposure conditions are limited. This reduces confidence regarding the long-term reliability of waste-derived materials in permanent ground support applications.
4. Weak mechanism-level linkages: Many studies report macro-scale performance without adequately linking observations to microstructural and chemical mechanisms. There remains a shortage of integrated investigations combining SEM/BSE, XRD, TGA/DTG, MIP, nanoindentation, and ITZ characterisation to quantitatively explain how hydration products, pore refinement, and interfacial interactions influence mechanical and durability behaviour.
5. Unclear replacement-level envelopes: Although moderate replacement levels frequently produce favourable results, transferable substitution limits have not been established across different applications, aggregate gradations, or tailings mineralogies. Frameworks capable of simultaneously optimising rheological, mechanical, durability, and sustainability performance remain underdeveloped.
6. Limited scale-up, standardisation, and field validation: Most available evidence is derived from laboratory-scale investigations. Pilot-scale and field-scale studies assessing rebound, dust generation, overbreak, spraying efficiency, and early load-bearing capacity remain limited. Furthermore, standardised testing methodologies for material characterisation, rheological assessment, spraying parameters, and curing conditions are required to improve comparability between studies.
7. Gaps in sustainability, economics, and logistics: Comprehensive life-cycle assessment (LCA) studies and cost–benefit analyses remain scarce. Factors including material processing requirements, transport distances, on-site beneficiation opportunities, and blending strategies have not been systematically incorporated into sustainability assessments. Material variability and regulatory acceptance also remain significant barriers to industrial implementation.
8. Environmental and compliance considerations for tailings-rich systems: For tailings-rich systems, particularly those incorporating bauxite residue (red mud), leachate behaviour and contaminant mobility remain insufficiently understood. The effectiveness of contaminant immobilisation under cracked, aged, or carbonated conditions requires further investigation. These factors are critical for environmental approval, regulatory compliance, and responsible deployment in environmentally sensitive environments.

To provide a structured framework for addressing the identified research gaps, Table 6 presents a comparative research roadmap linking priority research areas with the relevant material systems, recommended experimental methods, and desired engineering outcomes. This roadmap highlights key directions for advancing the understanding, validation, and implementation of waste-derived materials in shotcrete and cementitious systems.

## 5. Conclusions

This review synthesised findings from studies investigating the use of CGA, FA, and mining tailings in cementitious materials, with particular attention to their potential relevance for shotcrete applications. The reviewed literature demonstrates that these waste-derived materials can partially replace conventional aggregates and cementitious constituents while maintaining acceptable engineering performance when appropriately incorporated into cementitious systems.

The strongest and most reliable evidence was identified for CGA, particularly in relation to shotcrete applications. Across the reviewed studies, CGA generally maintained or improved workability and mechanical performance at moderate replacement levels when appropriate grading and mix designs were adopted. CGA also possesses the most extensive shotcrete-specific evidence base among the reviewed waste-derived materials, providing the highest level of confidence regarding practical implementation.

A moderate level of confidence was identified for FA. FA is a well-established SCM, with substantial evidence supporting improvements in workability, pore refinement, and durability performance. However, compared with conventional concrete applications, relatively fewer studies have investigated hydration mechanisms, microstructural evolution, and long-term durability specifically within FA-modified shotcrete systems.

The lowest level of confidence was associated with mining tailings. Although many studies report favourable mechanical and durability performance, results remain highly dependent on mineralogy, particle characteristics, processing history, and functional role within the cementitious system. Consequently, findings obtained for one tailing type cannot be readily generalised to others. Long-term durability assessments, mechanistic investigations, and shotcrete-specific validation also remain comparatively limited.

From a practical implementation perspective, CGA currently demonstrates the highest readiness for shotcrete applications, followed by FA, which benefits from extensive industrial experience but still requires further shotcrete-specific validation. Mining tailings show considerable future potential, particularly for resource recovery and waste utilisation, but their readiness remains dependent on source-specific characterisation, durability validation, and field-scale implementation.

Several important research gaps remain, particularly in relation to hybrid waste-material systems, rheology and pumpability characterisation, long-term durability assessment, mechanistic understanding, and field-scale validation. The comparative research roadmap presented in Table 6 provides a structured framework for addressing these priorities and advancing the implementation of waste-derived materials in shotcrete and cementitious systems. Addressing these challenges will support the development of evidence-based specifications, improve confidence in material selection, and facilitate the broader adoption of waste-derived materials in civil and mining infrastructure.

## Figures and Tables

**Figure 1:**
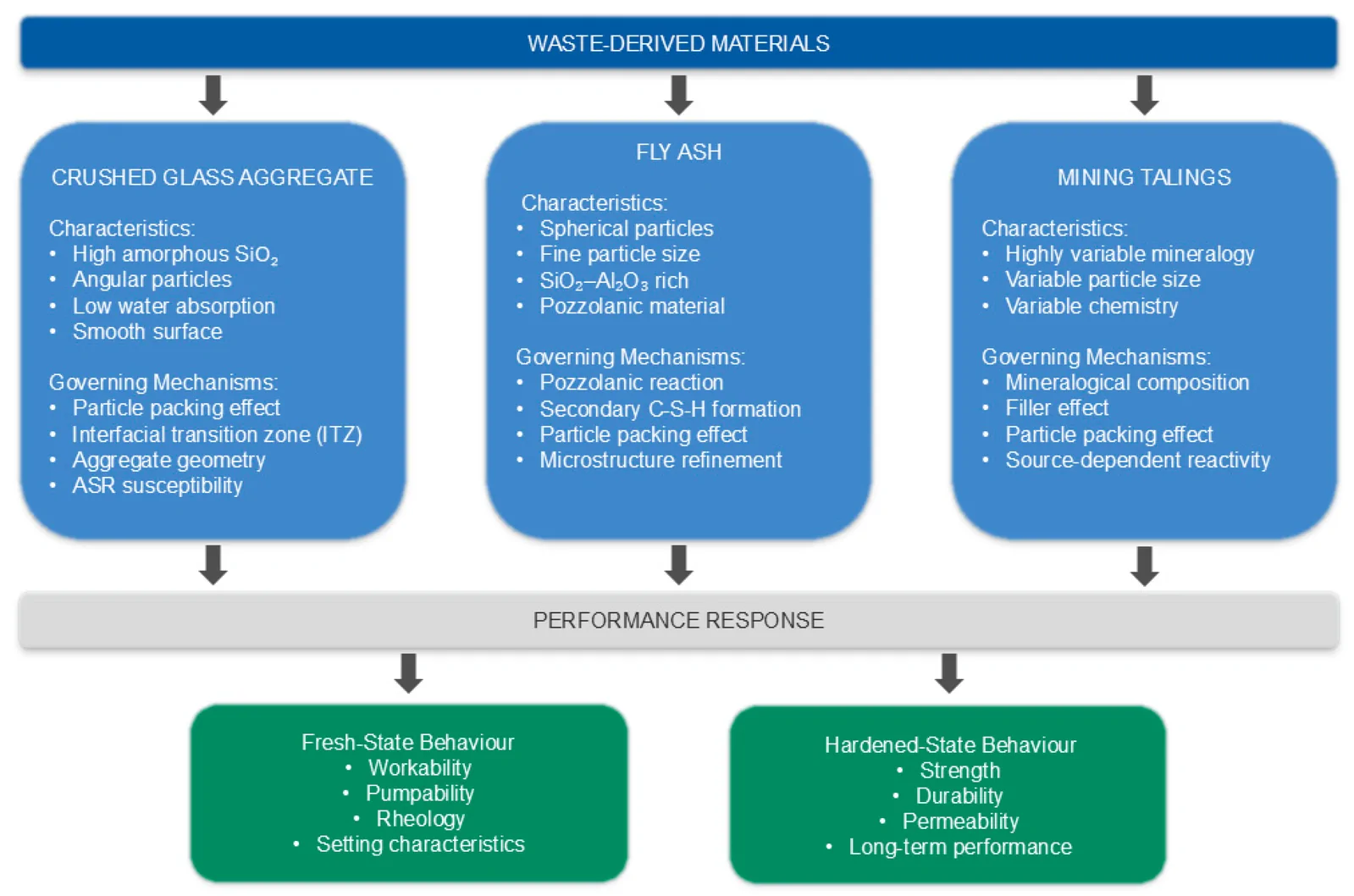
Material characteristics, governing mechanisms, and resulting fresh-state and hardened-state performance of crushed glass aggregate, fly ash, and mining tailings in cementitious systems. The figure was developed based on the synthesis of findings reported in previous reviews and representative studies on CGA [23,25,27], FA [18,29,30,31,32,33], and mining tailings [19,20,21,22,24].

**Table 1:** Comparative synthesis of the principal characteristics, performance trends, governing mechanisms, advantages, and limitations of crushed glass aggregate, fly ash, and mining tailings reported in the literature. Information synthesised from CGA studies [23,25,26,27,28,35,36,37,38,39,40,41,42,43,44,45,46,47,48,49,50,51,52,53,54,55,56], FA studies [18,29,30,31,32,33,57,58,59], and mining tailings studies [19,20,21,22,24,60,61,62,63,64,65,66,67,68,69,70,71,72,73,74,75,76,77,78,79,80,81,82,83,84,85,86,87,88,89,90,91,92,93,94,95,96,97,98,99].

Property	Crushed Glass Aggregate	Fly Ash	Mining Tailings
**Primary function**	Fine aggregate replacement	Supplementary cementitious material (SCM)	Aggregate replacement, SCM, or filler
**Typical replacement range (by functional role)**	10–30% commonly reported; higher levels possible with optimisation	5–20% commonly reported; up to 40% depending on application	Highly source-dependent
**Workability**	Mixed response; influenced by particle angularity, grading, and glass type	Generally improved due to spherical particle morphology	Variable; dependent on particle size, morphology, and mineralogy
**Pumpability**	Generally maintained or improved at moderate replacement levels	Generally improved due to enhanced flowability	Limited and source-dependent evidence
**Setting behaviour**	Limited influence; dependent on mixture composition	May delay setting and early hydration	Variable depending on mineralogical composition
**Compressive strength**	Often maintained or improved at moderate replacement levels; excessive content may reduce strength	Moderate replacement levels generally maintain performance; excessive contents may reduce early-age strength	Highly dependent on tailing type and replacement strategy
**Tensile/Flexural strength**	Mixed results; sensitive to particle bonding and replacement level	Generally maintained at moderate replacement levels	Variable and source-dependent
**Permeability**	Often reduced at moderate replacement levels due to matrix densification	Commonly reduced through pozzolanic reactions and pore refinement	Variable depending on particle characteristics and reactivity
**Chloride resistance**	Commonly improved at moderate replacement levels	Generally improved owing to refined pore structure	Source-dependent
**ASR behaviour**	Potential concern due to high silica content; mitigation may be required	Often contributes to ASR mitigation through pozzolanic reactions	Generally dependent on mineralogy and reactive constituents
**Durability performance**	Mixed but generally positive at moderate replacement levels	Commonly positive due to reduced permeability and improved microstructure	Highly variable and source-dependent
**Primary governing mechanisms**	Particle packing, aggregate geometry, ITZ characteristics, ASR susceptibility	Pozzolanic reaction, secondary C–S–H formation, particle packing, pore refinement	Mineralogical composition, filler effect, particle packing, source-dependent reactivity
**Main advantages**	Reduces natural sand consumption; abundant waste resource	Reduces cement consumption; established SCM with extensive industrial use	Large-scale waste utilisation and resource recovery
**Main limitations**	Grading sensitivity and potential ASR concerns	Variability in quality, declining availability, lower early-age reactivity	High variability, potential contaminants, source-specific behaviour

**Table 2:** Summary of representative studies on the utilisation of CGA in cementitious systems.

First Author (Year)	Replacement and Range	Key Findings
Bhat (2024) [39]	Aggregate and cement (5–25%)	Optimal replacements (15% coarse aggregate, 15% fine aggregate, and 10% cement) increased compressive strength by 22.1%, 20.1%, and 13.1%, respectively, while reducing water absorption.
Adaway (2015) [40]	Fine aggregate (15–40%)	Compressive strength improved up to 30% replacement; slump varied due to angular particle geometry; ASR risk mitigated by controlling particle size.
Jahami (2022) [36]	Fine aggregate (25–75%)	Workability improved with higher glass content; 50% replacement increased compressive strength by 27% and flexural strength by 50%.
Jiao (2020) [37]	Fine aggregate (25–100%)	Ultra-high-performance concrete achieved highest compressive strength at 75% replacement; fluidity improved with increasing glass content; recommends further ITZ mechanism studies.
Tamanna (2020) [28]	Fine aggregate (20–60%)	Optimal compressive strength at 20% replacement; 60% replacement enhanced chloride resistance but reduced workability.
Liu (2024) [55]	Aggregate (25–100%) + glass powder	25% glass aggregate provides best balance of sustainability, cost, and strength; 100% replacement significantly reduced porosity and improved durability.

**Table 3:** Summary of representative studies on the utilisation of FA in cementitious systems.

First Author (Year)	Replacement and Range	Key Findings
Win (2023) [29]	Cement replacement: 5–30%	FA reduced compressive strength at all levels; 5%, 15% and 20% showed the least reduction. Slump increased with FA content, and moderate FA contents (15–25%) improved flexural strength. Strength development slowed in cold climates.
Wang (2021) [30]	Cement replacement: 10–40%; varying fineness	Ultrafine FA (<5 μm) at 10–30% improved 28- and 60-day compressive strength due to increased pozzolanic activity. Finer particles increased slump, with an exponential relationship between fineness and workability.
Wu (2022) [31]	Cement replacement: 0–35% (shotcrete)	Increasing FA reduced compressive, tensile, and flexural strength. ≤15% replacement caused minimal strength reduction and maintained structural safety for shotcrete applications.
Lu (2018) [32]	Cement replacement: 10–40% (shotcrete)	Strength declined with higher FA content. Recommended limits: ≤15% for compressive strength, ≤10% for tensile strength, and ≤30% for flexural strength. Optimal FA ≤ 10% for structural shotcrete.
Song (2021) [57]	Predictive modelling of FA mixes	Machine learning models (Bagging Regressor: R^2^ = 0.95) accurately predicted compressive strength trends. Identified FA replacement and fineness as major determinants of mechanical performance.

**Table 4:** Summary of representative studies on the utilisation of mining tailings in cementitious systems.

First Author (Year)	Replacement and Range	Key Findings
Zhang (2020) [70]	Iron tailings sand replacing fine aggregate (20–100%) in UHPC	40% replacement produced the highest compressive strength; up to 80% showed comparable performance to control, indicating UHPC tolerates high IOT contents with minimal mechanical penalty.
Jayasimha (2022) [75]	Iron tailings as fine aggregate	40% replacement optimum; increased workability due to improved fineness modulus; compressive strength increased at all ages.
Ling (2023) [80]	Iron tailings aggregate (0–50%)	Slump and cohesion decreased as IOT increased; compressive strength increased to 40% (9.7% above control) before declining. Improved ITZ density attributed to rough tailings surface and local w/c reduction around particles.
Song (2024) [82]	Gold tailings replacing sand	At 30%, compressive and tensile strength increased; water absorption decreased; freeze–thaw and chloride resistance improved via enhanced pozzolanic activity (SiO_2_ and Al_2_O_3_).
Thomas (2013) [86]	Copper tailings replacing sand (0–60%)	All replacement levels produce acceptable strength and durability characteristics.
Chavan (2021) [67]	Bauxite (red mud) replacing cement (2–20%)	Optimal at 12%; increased compressive (16%), tensile (41%), and flexural (19%) strength; improved chloride resistance. High RM contents reduced slump due to fine particle size and high-water demand.

**Table 5:** Chemical compositions of selected mine tailings (wt.%), adapted from Gou et al. [20].

Mineral Type	Source Country	SiO_2_	Al_2_O_3_	Fe_2_O_3_	CaO	MgO	SO_3_	K_2_O	Na_2_O	LOI
Zn–Pb tailings	Tunisia	15.5	6.6	5.9	19.9	4.0	21.9	0.3	–	15.1
Zn–Pb tailings	China	69.9	10.4	1.9	2.2	1.4	0.6	2.2	0.5	3.7
Zn–Pb tailings	Morocco	68.4	9.4	2.2	2.0	0.5	0.4	5.5	0.7	–
Zn–Pb tailings	Serbia	43.3	11.1	15.6	20.0	4.3	0.3	1.0	0.9	5.6
Iron tailings	China	75.2	2.6	11.3	1.5	2.1	0.1	0.4	0.5	–
Iron tailings	Brazil	24.2	4.8	45.9	–	–	–	–	–	4.1
Iron tailings	Malaysia	56.0	10	8.3	4.3	–	–	1.5	–	3.3
Iron tailings	China	52.1	17.1	9.1	12.8	3.7	–	0.3	1.0	3.2
Copper tailings	India	75.0	12.2	3.6	0.2	0.5	–	1.9	4.3	2.1
Gold tailings	Finland	49.9	10.4	9.7	11.1	5.9	–	1.3	3.0	12.9
Bauxite tailings	China	32.2	37.4	8.7	3.2	0.9	–	–	–	13.7
Phosphate tailings	China	2.1	0.1	0.8	36.8	18.9	1.0	–	0.1	35.8
Quartz-based tailings	Korea	79.5	9.5	3.2	0.5	0.6	–	3.2	0.7	2.5

Note: LOI = Loss on Ignition. The symbol “–” indicates that the corresponding value was not reported in the original source.

**Table 6:** Comparative research roadmap linking key research priorities, material systems, recommended test methods, and desired engineering outcomes for waste-derived materials in shotcrete and cementitious systems.

Research Priority	Material(s)	Recommended Methods	Desired Engineering Outcome
Hybrid material systems	CGA–FA, FBMT–FA, tailings–FA	Comparative experimental programs incorporating rheology, mechanical and durability testing	Improved understanding of combined effects and mix optimisation
Rheology and pumpability	CGA, FA, tailings, hybrid systems	Rheometry, pump-loop testing, sprayability and rebound assessment	Reliable placement and operational performance in shotcrete
Durability performance	All materials	Long-term chloride, sulfate, carbonation, freeze–thaw and ASR testing	Improved confidence in service-life performance
Hydration and microstructural mechanisms	FA and mining tailings	SEM/BSE, XRD, TGA/DTG, MIP, nanoindentation and ITZ characterisation	Mechanistic understanding linking microstructure to engineering performance
Replacement-level optimisation	All materials	Multi-objective optimisation frameworks integrating fresh-state, mechanical and durability performance	Application-specific design guidelines
Field validation and standardisation	All materials	Pilot-scale and field-scale shotcrete trials	Increased technology readiness and implementation confidence
Sustainability and economics	All materials	Life-cycle assessment (LCA) and cost-benefit analysis	Quantification of environmental and economic impacts
Environmental compliance	Tailings-rich systems	Leachate testing, contaminant mobility assessment and long-term exposure studies	Improved regulatory acceptance and environmental assurance

## Data Availability

No new experimental data were created or analysed in this study. Data sharing is not applicable to this article.

## Supplementary Materials

The following supporting information can be downloaded at https://www.mdpi.com/article/10.3390/ma19194049/s1, Supplementary Material S1: Literature review matrices (Tables S1–S3), including study characteristics, replacement strategies, experimental design parameters, and key findings. References associated with the supplementary tables are also provided. Table S1: Literature review matrices regarding the utilisation of crushed glass aggregate; Table S2: Literature review matrices regarding the utilisation of crushed glass aggregate fly ash; Table S3: Literature review matrices regarding the utilisation of crushed glass aggregate mining tailings in cementitious composites.

## Abbreviations

The following abbreviations are used in this manuscript:

AFm	Monosulfoaluminate
AFt	Ettringite
ASR	Alkali–Silica Reaction
CGA	Crushed Glass Aggregate
CH	Calcium Hydroxide
CS	Copper Slag
C–S–H	Calcium Silicate Hydrate
EDS	Energy Dispersive Spectroscopy
FA	Fly Ash
FBMT	Fine Bauxite Mining Tailings
FTIR	Fourier Transform Infrared Spectroscopy
HEDP	1-Hydroxyethylidene-1,1-Diphosphonic Acid
IOT	Iron Ore Tailings
ITP	Iron Ore Tailings Powder
ITS	Iron Ore Tailings Sand
ITZ	Interfacial Transition Zone
LCA	Life-Cycle Assessment
LOI	Loss on Ignition
MCDA	Multi-Criteria Decision Analysis
MIP	Mercury Intrusion Porosimetry
MOS	Magnesium Oxysulfate Cement
NMR	Nuclear Magnetic Resonance
OPC	Ordinary Portland Cement
RA	Recycled Aggregate
RCA	Recycled Coarse Aggregate
RCPT	Rapid Chloride Penetration Test
RM	Red Mud
SCM	Supplementary Cementitious Material
SEM	Scanning Electron Microscopy
TGA	Thermogravimetric Analysis
TSF	Tailings Storage Facility
UHPC	Ultra-High-Performance Concrete
WFS	Waste Foundry Sand
WGP	Waste Glass Powder
XRD	X-ray Diffraction
