# Supplementary material for: Comparative Assessment of Crushed Glass Aggregate, Fly Ash, and Mining Tailings in Cementitious Composites and Shotcrete Applications
Source: Materials (Basel). 2026 Sep 22;19(19):4049. doi: 10.3390/ma19194049 (PMC13666325; doi:10.3390/ma19194049)
Supplement: Supplementary file 1 [file materials-19-04049-s001.zip › materials-4575090-supplementary.pdf]

Supplementary Material S1

S1

Table S1. Literature review matrices regarding the utilisation of crushed glass aggregate.

First Author	Year	Ref #	Study Type	Application	Substitution	Exp design	w/c	Findings
Bhat	2024	[39]	Primary Experimental Study	Concrete	Glass Particles	Replacing aggregate and cement at 5%, 10%, 15%, 20% and 25% by weight.	-	Results demonstrated that the optimum replacements were 15% for both coarse and fine aggregate, and 10% for cement. This enhanced compressive strength by 22.1%, 20.1%, and 13.1%, decreased flexural strength by 4.43%, 6.8%, and 2.85%, and decreased tensile strength by 11.3%, 8.36%, and 8.96%, respectively. Water absorption decreased to 3.83%, 4.23%, and 4.41%, compared to 4.61% for the control. Comparable results were observed with 20% replacements for coarse and fine aggregate, and 15% for cement.
Song	2025	[43]	Primary Experimental Study	Concrete	Recycled aggregate and waste glass powder	Evaluating microstructure, mechanical strength, and bonding performance of concrete utilising waste glass	-	The research tailored two testing regimes: natural aggregate with variations in waste glass powder, and recycled aggregate (waste concrete) with variations in waste glass powder. The PSD for the materials mimicked the gradations for natural and recycled aggregates, and ordinary Portland cement and waste glass powder. Aggregate was

						powder at 20%, 40% and 60%, replacing cement.		replaced by volume and cement by weight, with a w/b ratio of 0.5. Recycled aggregate was added in a saturated surface-dry condition (SSD) to mitigate higher water absorption. River sand and water content were constant, with superplasticiser dosage adjusted for workability. Results showed that increasing waste glass powder replacement decreased compressive and splitting tensile strength at 28 days, but strength gains were observed from 28 to 90 days due to secondary C-S-H gel formation and pore structure refinement. Higher waste glass replacements decreased bonding strength with steel reinforcement, but the pull-out failure mode remained consistent, indicating aggregate type determines failure mode.
Adaway	2015	[40]	Primary Experimental Study	Concrete	Recycled glass replacing fine aggregate	Evaluating the compressive strength of structural concrete utilising 15%, 20%, 25%, 30%, and 40%	0.41	Glass fine aggregate particles > 1.18mm were discarded to avoid excessive ASR. Slump test results for mixes with waste glass varied from 15% above to 30% below the reference mix. Ismail et al. (2009) cited in [40] observed similar trends, attributing them to poor glass geometry and reduced fineness modulus. The mix design accounted for this by altering the coarse-to-fine aggregate ratio. Discrepancies were ascribed to angular geometry reducing available cement paste, thus reducing mix fluidity. Compressive

								strength increased with waste glass inclusion up to 30%, achieving 9% and 6% higher at 7 and 28 days, due to increased bonding strength of angular glass particles with cement paste.
Jahami	2022	[36]	Primary Experimental Study	High-Strength Concrete	Waste glass as fine aggregate	Replacing natural sand for waste glass sand at 25%, 50% and 75%	-	Workability of the concrete improved with increased waste glass sand content. The optimal dose of waste glass sand was 50%, resulting in increases of 7% in elasticity modulus, 27% in compressive strength, 9% in splitting tensile strength, and 50% in flexural strength.
Jiao	2020	[37]	Primary Experimental Study	Ultra-high-performance concrete	Waste glass sand as fine aggregate	Replacing quartz sand and river sand with waste glass at 25%, 50%, 75%, and 100%.	-	Waste glass sand improved compressive strength, especially at a 75% replacement level. It also significantly enhanced fluidity as the replacement ratio increased. Future research is suggested to investigate the intrinsic mechanisms of interface behaviour with waste glass and the performance after fire or blasting.
Gerges	2018	[44]	Primary Experimental Study	Concrete	Waste glass as aggregate	Evaluating waste glass replacing natural aggregate. Coarse aggregate substitution at 33%, 50%, 67%, 100%, and fine	-	Using recycled glass as a 100% replacement for sand resulted in minimal changes: a slight reduction in fresh density, an insignificant increase in air content, and a 10mm decrease in slump compared to the control. Compressive strength test results showed a

						aggregate at 100%.		15.8% and 17.1% decrease at 7 and 28 days, respectively, compared to the control.
Mohammed	2021	[41]	Primary Experimental Study	Concrete	Waste glass powder and waste fine plastic aggregate	Evaluating concrete incorporating glass waste replacing 15% of cement. Crushed waste plastic replacing 10% and 20% fine aggregate.	0.32	Evaluating the results with glass powder as the sole variable, the experimental results compared to the control showed a 12.5% decrease in slump, a 2.61% increase in density, a 7.46% increase in compressive strength for cylindrical samples, a 14.1% increase in splitting tensile strength, a 1.7% increase in flexural strength, and a 6.01% increase in elastic modulus.
Ahmad	2023	[49]	Primary Experimental Study	Self-compacting concrete	Demolition waste as coarse aggregate and recycled crushed glass as fine aggregate.	Utilising demolition waste at 50% and 100% and recycled crushed glass at 10% and 20%.	-	Workability decreased for recycled coarse aggregate but improved with recycled crushed glass, due to their respective higher and lower water absorption. Compressive, flexural, and tensile strength, as well as ultrasonic pulse velocities, significantly increased at 56 days for samples with recycled crushed glass. The greatest strength was achieved by incorporating 20% glass.
Al-Kizwini	2020	[42]	Primary Experimental Study	Concrete	Plastic and glass waste	Evaluating the reuse of plastic, glass waste, and combined plastic and glass. Substituting sand	-	Slump and compressive strength increased with higher waste glass content, with 20% and 15% replacement of sand and cement, respectively, attributed to the silica content.

						and cement at 5%, 10%, 15%, and 20%.		
Asa	2019	[56]	Primary Experimental Study	Mortar	Crushed waste glass replacing fine aggregate	Evaluated the compressive and splitting tensile strength behaviour of mortar containing crushed waste glass at 5%, 10%, 15%, and 20% replacing sand by volume, with highly reactive metakaolin (HRM) partially replacing cement, both with and without water-reducing admixtures.	-	Evaluating four sets of experimental tests: the control, mixes with cement only and glass aggregate substitution, mixes with 12% mineral admixtures (HRM) partially replacing cement with glass replacements (w/c ratio 0.58), and three additional mixes. The additional mixes included: 1.2% WRA by weight of cement (w/c ratio 0.45), 20% glass aggregate with 1.2% plasticiser (w/c ratio 0.45), and 12% HRM replacing cement by weight, 20% glass aggregate, and 1.2% plasticiser (w/c ratio 0.5). Results showed that with a constant w/c ratio of 0.54, increasing crushed waste glass content decreased density and strength, though 20% glass increased later age strength. HRM and plasticiser improved compressive strength in mixes with crushed waste glass.
Steyn	2021	[27]	Primary Experimental Study	Concrete	Waste crushed glass as fine aggregate	Studied concrete utilising crushed clear flat glass at 15% and 30%, replacing natural	-	The glass decreased workability, increased air content, and improved mechanical and durability performance of the concrete. It was recommended that due to the undesirable workability properties of the concrete mixes utilising glass, chemical admixtures should

						fine aggregate by volume.		be used to improve the workability of conventional concrete.
Tamanna	2020	[28]	Primary Experimental Study	Concrete	Recycled waste glass as fine aggregate	Utilising 3mm recycled waste glass as coarse sand for fine aggregate replacement at 20%, 40% and 60%.	-	Results showed that waste glass significantly impacted workability, reducing slump and density as replacement levels increased. The optimal replacement for compressive strength was 20%, increasing it by 5.8%. There was no significant difference in flexural and tensile strength between the control and waste glass mixes. A 60% replacement enhanced resistance to chloride ion penetration, and coarse sand replacement reduced ASR due to the pozzolanic reaction with alkali during cement hydration. It was concluded that 60% recycled crushed glass can be used in concrete, but additional admixtures should be employed to mitigate reduced workability.
Ishaq	2025	[35]	Primary Experimental Study	Concrete	Waste glass powder as a cement replacement	Analysing the effect of chemical compounds in cement and waste glass powder using mono-variate, bi-variate, and multi-variate approaches.	-	Based on the evaluation, in the mono-variate state, compressive strength improves with the presence of silicon dioxide, with the optimal glass replacement being 30%. However, an increase in aluminium oxide content results in a decrease in compressive strength. It was concluded that to improve compressive strength while maintaining workability and mechanical performance, glass powder

								replacement levels ranging from 10% to 15% are recommended.
Liu	2024	[55]	Primary Experimental Study	Eco-pervious concrete	Waste glass as aggregate and powder	Evaluating crushed waste glass aggregate (2.36-5mm) at 25%, 50%, 75%, and 100%. Glass powder replacing cement at a constant 88kg/m³ throughout the eco mixes.	-	The study employed integrated life cycle assessment and multi-criteria decision analysis to optimally utilise construction and demolition (C&D) waste and waste glass (WG) in concrete. Using the VIKOR method to identify optimal concrete mixtures, it was found that 25% WG substitution provided the best balance between sustainability, cost-effectiveness, and mechanical integrity. Significant environmental benefits were achieved by utilising 100% WG aggregate and 16.7% WG powder, reducing energy consumption by 573 MJ and carbon dioxide emissions by 73 kg.

Rahma	2017	[45]	Primary Experimental Study	Concrete	Glass powder	The first campaign investigated glass powder incorporation from 2.5% to 15% in 2.5% increments without superplasticiser. The second campaign used superplasticiser at 0.96 L/100 kg cement and evaluated four mixes: a control mix, a mix with 25% water reduction, a mix with 25% water reduction plus 7.5% glass powder, and a mix with 30% water reduction plus 7.5% glass powder.	-	They emphasis the significance of using fixed quantities of cement to distinguish the effects of glass powder from those of cement. The results showed that for normal concrete without superplasticiser, the workability decreases as the amount of glass powder increases. This was attributed to the fineness and high surface area of the glass powder. In the second campaign, using glass powder with plasticiser allowed for a reduction in the amount of water needed, thereby increasing the strength while improving workability.

Sathiparan	2024	[25]	Review Article	Pervious concrete	Crushed waste glass and glass powder	Potential use in sustainable pervious concrete.	-	The review article highlights the pozzolanic activity of glass powder and the challenges of adopting high levels of glass powder replacement levels. At low to moderate replacement levels, compressive, flexural strength, and durability properties improve due to the pozzolanic activity of the glass. However, at high levels, this trend reverses, increasing susceptibility to freeze-thaw cycles.
Wei	2024	[38]	Primary Experimental Study	Concrete	Recycled glass granules replacing natural river sand.	Investigating the effect of glass granules on rebar corrosion and the loss of bond between rebars and concrete due to accelerated corrosion. Replacing natural river sand for glass granules at 25%, 50%, 75%, and 100%.	0.5	High replacement rates effectively reduced concrete porosity. Glass granules improved corrosion resistance and extended rebar corrosion periods due to optimised concrete microstructure. Compressive strength increased with age, with 25% replacement achieving superior strength. Increased substitution reduced porosity, attributed to high SiO_2 content in glass aggregate reacting with $Ca(OH)_2$ to form additional C-S-H gel, densifying the pore structure. A 100% replacement exhibited 71% relative porosity compared to the control. Glass granules slowed bond strength reduction, with 25% inclusion achieving the greatest peak bond strength at all corrosion exposure times. All substitution levels at 48 hours of corrosion

								exposure achieved greater peak bond strength compared to the control.
Huang	2025	[46]	Primary Experimental Study	Concrete	Waste glass	Investigated machine learning systems for efficient use of waste glass in concrete to maximise carbon credits towards net zero emissions.	-	Six machine learning models - Gradient Boosting Regressor (GBR), Random Forest (RF), Adaptive Boosting (AdaBoost), Support Vector Regression (SVN), Deep Neural Network (DNN), and k-Nearest Neighbours (kNN) - were used to predict compressive strength, ASR, and saved carbon credits. The influential feature for prediction was age, AL_2O_3C content, and waste glass replacement. Among the models evaluated, GBR and SVR achieved superior prediction performance with R^2 values of 0.95, 0.97, and 0.99 for compressive strength, ASR, and saved carbon credits, respectively.
Kazmi	2021	[26]	Primary Experimental Study	Geotechnical applications	Crushed waste glass	Evaluated the potential of crushed waste glass as an alternative to natural and manufactured sand by comparing the three materials through	-	They concluded that the geotechnical behaviour of crushed waste glass at a given particle size and gradation are similar, and sometimes superior, to natural and manufactured sand, with silica being the dominant mineral. The comparable behaviour between glass and natural sand was due to similar chemical composition, specific gravity, and angular particle shape. Crushed waste glass exhibited the highest permeability and abrasion resistance. Shear

						geotechnical, mineralogical and morphological analysis.		strength tests revealed a greater friction angle when saturated compared to dry conditions, suggesting enhanced stability when saturated.
Disfani	2012	[47]	Primary Experimental Study	Road applications	Recycled crushed glass	Examined the environmental risks of using fine and medium recycled crushed glass to replace natural aggregates in road works and geotechnical fill.	-	Evaluating the total and leachate concentration, results show that both fill materials fall well below the acceptable limits and pose no hazard to surface or ground water.
Kazmi	2020	[23]	Review Article	Concrete	Crushed waste glass as a SCM and aggregate.	Waste glass in civil engineering applications.	-	Major findings highlight that fineness and substitution level determine the behaviour of crushed waste glass used as a SCM. Using waste glass as aggregate decreases workability due to mechanical interlocking from its angular nature [37, 40] cited in [23]. Studies show mixed results on strength and ASR triggers, attributed to particle size and chemical composition. ASR expansion can be mitigated by adopting the critical particle size where ASR does not occur, Bignozzi et al. (2015), Bazant et al. (2000), cited in [23].

Ali	2012	[48]	Primary Experimental Study	Self-compacting concrete	Recycled glass as fine aggregate	Examined partial replacement of sand at 10%, 20%, 30%, 40%, and 50%, with varied cement contents 300, 400, and 450 kg/m³	0.4	Results showed that as recycled glass content increases, slump flow increases, while strength properties and static modulus of elasticity decrease. The decrease in strength is attributed to the high smoothness of waste glass, which reduces bond strength with the cement paste, leading to cracks and weak adhesion.
Wang	2022	[54]	Primary Experimental Study	Structural concrete	Washed and unwashed glass fines replacing fine aggregate	Evaluation of glass fines partially replacing sand at 10%.	0.53	Unwashed glass fines alone are unsuitable as fine aggregate, but mixing 10% with natural sand dilutes contamination to acceptable levels. Washed glass fines passed all contamination tests. Slump was identical for control and washed glass mixes, with a slight reduction for unwashed mix. Both unwashed and washed glass fines showed less than 2% reduction in compressive strength compared to the control. The 10% inclusion did not critically affect ASR due to 25% FA in cement mixes, which suppresses ASR expansion as cited by Du et al. (2013) cited in [43].

S2

Table S2. Literature review matrices regarding the utilisation of fly ash.

First Author	Year	Ref #	Study Type	Application	Substitution	Exp design	w/c	Findings
Win	2023	[29]	Primary Experimental Study	Shotcrete	FA	FA substituting GP cement. FA adoption at 5%, 10%, 15%, 20%, 25%, and 30%.	-	FA decreased compressive strength at all levels, with better performance at 5%, 15%, 20%. Compared to the control, compressive strength decreased by 2.3%, 9.4%, and 15.1%, slump increased by 0%, 5.7%, and 9.5%, respectively. Moderate FA contents (15-25%) improved 28-day flexural strength. FA adoption led to longer drying and curing times, and slower strength development in cold climates.
Rosenberg	2010	Cited in [29]	Secondary Citation	Concrete	FA	FA in concrete	-	Samples containing 20% FA reached 75% of the control strength in the ASTM C109 test, meeting the 7- or 28-day strength requirements of ASTM C618. FA particles, being spherical and similar in size to Portland cement, reduce the water needed for mixing concrete, improving workability in precast concrete and resulting in sharper corners, edges, and better surface appearance.

Wang	2021	[30]	Primary Experimental Study	Concrete	FA	Study of fineness and dosage of FA in concrete. Analysing three different particle sizes and different dosages 10%, 20%, 30%, and 40%.	-	10% and 30% FA in concrete with particles <5um improve 28-day and 60-day compressive strength due to increased pozzolanic activity. The highest bending and splitting tensile strength occur at 20%-30%. As ultrafine FA increases, slump (workability) also increases but at a decreasing rate. Smaller particle sizes result in a greater slump, with an exponential relationship between particle size reduction and slump.
Wu	2022	[31]	Primary Experimental Study	Shotcrete	FA	FA content in shotcrete. Content variations from 0% to 35% at 5% intervals.	-	As FA content increases, strength decreases. High levels reduce compressive, tensile, and flexural strength. It was concluded that ≤15% shows minimal decreases, and maintaining the level ensures adequate strength for structural safety.
Lu	2018	[32]	Primary Experimental Study	Shotcrete	FA	Influence of FA content on mechanical properties. Replacing cement from 10% to 40% at 5% intervals.	-	Results demonstrate a decrease in strength as FA content increases, concluding that adoption should not exceed 15%, 10%, and 30% for compressive, splitting tensile, and flexural strength, respectively. Optimal FA content in shotcrete should not exceed 10% to minimise strength loss and ensure structural safety.

Song	2021	[57]	Primary Experimental Study	Concrete	FA	Prediction performance of machine learning algorithms to predict compressive strength of concrete containing FA	-	Focusing on comparing the prediction performance of various machine learning algorithms, they concluded that the bagging regressor (ensemble method) is the best, with a R^2 value 0.95, indicating it is the most accurate model among those compared. Machine learning techniques such as gene expression programming (GEP), artificial neural network (ANN), and decision trees (DT) demonstrate strong relationships between targeted and predicted strength properties, R^2 values of 0.86, 0.81, and 0.75, respectively.
Reshma	2021	[58]	Primary Experimental Study	Concrete	Untreated waste foundry sand and fly ash	Evaluated mechanical and fresh properties with 30% FA replacing cement and foundry sand at 10%, 20%, 30%, and 40%, replacing natural river sand for M40 concrete	-	Test results indicated that concrete quality increased with a 30% replacement of foundry sand, improving both mechanical and fresh properties. At 40% replacement, there were minimal strength reductions.

Panesar	2020	[18]	Review Article	Concrete	Limestone filler compared to SCM's (fly ash, slag cement, silica fume or metakaolin)	Examined early age hydration, mechanical properties, permeability, durability, and environmental impacts of limestone filler compared to SCM's in cement-based materials.	-	Comparing limestone filler to those containing low-calcium fly ash (class F), high-calcium fly ash (class C), silica fume, slag cement, and metakaolin. They concluded that limestone filler accelerates early-age hydration of cement, especially with fine particles. At less than 10% replacement, it enhances compressive strength and reduces chloride permeability. However, higher replacement levels decrease strength and increase porosity and permeability. While SCMs up to 20% replacement improves strength and reduces porosity and permeability. Limestone filler offers similar carbonation resistance to those containing SCMs but is less effective against sulfate attack, freeze/thaw action, and Alkali-Silica Reaction (ASR) at the same replacement level. At ≤10% replacement, limestone filler can match or exceed the performance of 100% Portland cement, resisting durability issues. Both limestone filler and SCMs lower the global warming potential of cement-based materials.
Sun	2023	[33]	Primary Experimental Study	Shotcrete	-	Impact of water-binder ratio, fly ash content, steel fiber content, and water-	0.38	The w/c ratio significantly impacts compressive strength. FA improves workability, shotcrete performance and strength development. At 15% FA, the concrete had the lowest permeability. The optimum superplasticiser dosage was 0.8%,

						reducing agent dosage.		higher amounts caused bleeding and poor aggregate encapsulation, while lower amounts decreased flowability, impacting shotcrete performance. Flexural and tensile strength are significantly affected by steel fibers, with the optimum dosage being 45kg/m3.

S3

Table S3. Literature review matrices regarding the utilisation of mining tailings in cementitious composites.

**First Author**	**Year**	**Ref #**	**Study Type**	**Application**	**Tailings / Sub**	**Exp design**	**w/c**	**Findings**
Zou	2023	[59]	Primary Experimental Study	Shotcrete	Iron	Ratio parameter optimisation of TA (waste rock and tailings sand) + FA and mechanical properties. Investigated 4 levels, with various: cement content, mass concentration, waste rock	-	Optimal ratio: cement content 290kg/m³, mass concentration 79%, waste rock 70%, fly ash 16%. Particle grading of waste rock and tailing sand significantly influences the strength and performance of shotcrete. Increasing the cement content or mass concentration reduces the formation of voids within the mixture, allowing a greater quantity of C–S–H hydration products to occupy the space between particles. This results in a denser skeletal structure and, consequently, improved macroscopic mechanical properties.

						content, fly ash content.		
Liu	2011	Cited in [72]	Secondary Citation	Shotcrete	Iron	IOT sub for natural sand	-	20% sub for sand has superior compressive strength than 100% natural sand
Zhang	2020	[70]	Primary Experimental Study	Ultra-High-Performance Concrete (UHPC)	Iron	IOT sub for sand in UHPC at 20%, 40%, 60%, 80% & 100%	-	40% highest comp strength, ≤80% IOT comparable results with control
Shi	2024	[71]	Primary Experimental Study	UHPC	Iron	IOT powder for cement, IOT sand for quartz sand	-	20% IOT powder + 50% IOT sand demonstrated highest compressive strength and flexural strength for mixes containing combined IOT powder and IOT sand
Liang	2022	[72]	Primary Experimental Study	Magnesium oxysulfate cement (MOS)	Iron	Optimising 1-hydroxethylidene-1, 1-diphosphonic acid (HEDP) and IOT sub for light-burned magnesia powder at 10%, 20%, 30%	-	Grinding IOT for 1hr improves reaction activity, HEDP forms a stable chelate (bonding of ions and the molecules to metal ions) with ((Mg (OH)(H_2O)) produced in the hydration of MgO. The setting time of MOS cement is delayed by slowing down the hydrolysis of active MgO to Mg $(OH)_2$. Optimal dosage is 0.75% HEDP, 10% mechanically activated IOT, which does not reduce the compressive strength of the MOS cement.

Lv	2019	[73]	Primary Experimental Study	Concrete	Iron	Complete replacement of natural aggregate with both fine and coarse Tailing Aggregate (TA) and FA	0.45	Better results that control with fixed w/b ratio (hardened state), Similar results for TA and NA with fixed approx. cement consumption
Yang	2020	[74]	Primary Experimental Study	Concrete	Iron	Iron tailing powder (ITP) as concrete admixture	-	Optimal range 450 m2/kg ~ 550 m2/kg, ≤ 30% replacement. ITP acts as physical filling, chemical activation has little effect on the activity of the powder.
Jayasimha	2022	[75]	Primary Experimental Study	Concrete	Iron	Sub as fine aggregate	-	40% optimal, increase in workability attributed to fineness modulus, increased compressive strength at all ages.
Vescovi	2023	[76]	Primary Experimental Study	Concrete	Iron	IOT sub for cement at 10% and 20% with three variations in IOT chemical composition	-	High Fe content even at 80% clinker for sample F20 closely matches compressive strength of the reference. IOT used composed almost entirely of Fe2O3, SiO2 and Al2O3, Iron Oxide, Silicon Dioxide and Aluminium Oxide, respectively. Gain in strength attributed to residual iron oxide however further studies are needed that only vary iron oxide content of IOT to determine the reason for the increase in strength as the interaction between pozzolanic and cementitious material is complex.

Han	2022	[77]	Primary Experimental Study	Concrete	Iron	IOT Powder to replace Class F FA at 0%, 15%, 25%, 35% & 50% with 0.38 w/b ratio and 0%, 10%, 17%, 25% & 35% with 0.28 w/b ratio	0.38 w/b, 0.28 w/b	50% sub mimics compressive strength results of reference, increased resistance to sulphate attack at 0.28 w/b. Suggested that decreasing below 0.28 w/b could eliminate adverse effects of high substitution ratio of ITP in FA concrete.
Bezerra	2021	[78]	Primary Experimental Study	Concrete	Iron	IOT (sludge and dry) sub for cement at 0%, 5%, 10%, and 15% in weight.	0.45 w/b	5% IOT sludge was found optimal resulting in a 5.37% decrease at 28-day compressive strength and a 5.76% increase at 90 days.
Cheng	2020	[79]	Primary Experimental Study	Concrete	Activated Siliceous iron	Partially replace cement at 0%, 10%, 20%, 30%, 40%.	-	Tailings pulverised for 3.5 hours. 30% sub demonstrated increased impermeability, frost resistance. Carbonation resistance was sub-standard at all levels compared to reference however meets requirements of practical construction works.
Ling	2023	[80]	Primary Experimental Study	Concrete	Iron	Evaluation under uniaxial cyclic compression for concrete utilising IOT aggregate. Control 0%, 20% IOT, 25% IOT,	-	IOT Aggregate 0.16 to 5.0 mm. Results demonstrated that IOTs crushed stone decreases slump and cohesion as content increases. Compressive strength increases as content increases up to 40% IOT content, 9.7% higher than control, then decreases. IOT as an aggregate: rough surface, high

						30% IOT, 40% IOT, 45% IOT, 50% IOT. IOT Content % is the ratio between the mass of all IOTs in concrete and the mass of the concrete test block.		relative surface area compared to sand and stone, large bonding force formed between ore and cement. IOT ore reduces the local water cement ratio inside the concrete and forms a hard cement shell around the ore improving the compressive strength when subjected to axial load. Decreasing IOT particle size can reduce the formation and fracture of the ITZ.
Yu-yun	2021	[81]	Primary Experimental Study	Shotcrete	Gold	Fine and Coarse aggregate replacement	-	Suitable for complete replacement
Widojoko	2014	[83]	Primary Experimental Study	Shotcrete	Gold	Fine aggregate replacement	-	25% sub resulted in an increase of compressive strength by 9%, and with Polcon as an admixture an increase of 11.7% compared to the control. Future research should include Polcon, or another added admixture with higher doses to increase strength.
Song	2024	[82]	Primary Experimental Study	Concrete	Gold	Replace river sand	-	30% replacement enhances comp and tensile strength, reduces water absorption, increases resistance to freeze-thaw cycles and chloride ion permeability. Attributed to particle size and pozzolanic activity (altered active $Si0_2$ and Al_2O3 content in the mix.

Lilies	2014	Cited in [82]	Secondary Citation	Shotcrete	Gold	Partial replacement of river sand	-	25% replacement resulted in 32.7 MPa, increased 28-day strength by 9% compared to reference. Gold tailings improve durability by increasing resistance to carbonisation and chloride ion penetration.
Luo	2022	[84]	Primary Experimental Study	Ultra-High-Performance Concrete (UHPC)	Gold	Influence of curing regimes (20°C water curing 28 days, 90°C water curing 2 days, 200°C dry air heating for 2 days, and combined curing 90°C water for 2 days and 200°C dry air heating for 2 days) on mechanical and microstructure of UHPC containing 60% gold tailings replacement for sand.	0.18	Mix proportions (kg/m³): Cement 794, Fly Ash 140, Silica Fume 233, Superplasticizer 14.7, Water 210, Sand 969. Results demonstrated that the strength of UHPC with 60% gold tailings were equal or slightly higher than the control under single curing regimes. Heat curing regimes increase strength, from hot water to hot air to combined heat curing. The only regime where the control gained a higher strength was the combined curing attributed to the reactivity of the gold tailings being weaker than quartz sand. Gold tailings SiO_2 content was 72.4% compared to quartz sand.

Garcia-Troncoso	2022	[85]	Primary Experimental Study	Concrete	Gold	River sand was replaced with gold mining tailings sand at 5%, 10%, 20%, 40%, 100%.	0.38	5% replacement achieves similar compressive strength results to conventional concrete. As levels increase so too does slump but decreases compressive strength.
Thomas	2013	[86]	Primary Experimental Study	Concrete	Copper	M25 concrete with copper tailings partially replacing river sand (0 - 60%),	0.4, 0.45, 0.5	0 - 60% sub for sand is suitable (all levels), exhibiting good strength and durability characteristics
Sambangi	2021	[100]	Primary Experimental Study	Concrete (self-compacting)	Copper and FA	FA to partially replace cement at 5%, 10%, 15%, 20%, 25%, and 30%. Copper slag sub for fine aggregate at 10%, 20%, 30%, 40%, 50%, 60%, 70%, 80%, 90%, and 100%	-	Optimal use of FA was 20%, demonstrating increased strength compared to the control and the other levels. Adopting the 20% FA and including copper slag, the optimal substitute value was 40% demonstrating the greatest compressive and 60% demonstrating the highest tensile strength.
Xiao	2020	[89]	Primary Experimental Study	Shotcrete	Coal	100% coarse aggregate with hybrid fine aggregate	0.45	100% sub of coarse aggregate, ≥12.5% river sand to allow for adequate workability. Linear fit of slump (increase % increase slump). Strength results were noted to be lower than other studies as they examined

						(tailing and sand)		partial replacement in the production of concrete.
Sun	2023	[90]	Primary Experimental Study	Shotcrete	Coal	Replace coarse aggregate and predict mechanical properties using machine learning	-	Governing variable affecting compressive strength is coal gangue aggregate, higher coal to sand ratio results in lower strength. Superior prediction performance and generalisation from PSO-BPNN and PSO-SVM. Optimum value regarding compressive strength was coal gangue size 100mm, 40% sub for sand, PVA fiber ratio %wt. to cement 0.005, sand ratio 1.32, the optimum design resulted in a 54% decrease.
Qiu	2021	[91]	Primary Experimental Study	Concrete	Coal at 40% with variant FA %	Replacement of natural aggregate with Tailing Aggregate (TA) and FA 0%, 10%, 20%, 30% & 40%	-	30% FA sub for cement with 0.42 w/c ratio best for strength and workability. FA at 30% led to an increased interfacial bond force, attributed to transformations in ITZ structure by microaggregates filling larger pores, reducing pores and crack width.
Qiu	2017	Cited in [91]	Secondary Citation	Concrete	Coal	Substitute for natural gravel at 0%, 20%, 40%, 60%. Study frost resistance.	0.42	40% substitution optimal via equivolumetric method to replace natural gravel.

Ikotun	2022	[22]	Review Article	Concrete	Iron, copper, Gold	Replacement for sand	-	Iron and gold tailings lower workability and that copper increases workability. Iron and gold give suitable compressive strength up to a certain %. Conflicting results for recommended max % of copper tailings in concrete (70%, 80%, 30%, 60%). Iron, copper, and gold share similar trends tensile strength, gold and copper impact on flexural strength are also similar, iron tailings demonstrate reduced performance for flexural strength. Standardised tests were recommended to ascertain a uniform trend on the mechanical properties from the use of mining tailings and that there are limited studies on the durability performance of concrete containing tailings.
Gou	2019	[20]	Review Article	Cement, Concrete	Zn-Pb, Iron, Copper, Gold, Bauxite, Phosphate, Quartz-based	Tailings in cement and concrete	-	Recommended that tailings should be ≤30% substitute as fine aggregates. Utilisation reduces workability. Tailings as cement replacement has negative effects on compressive strength due to low pozzolanic activity. Not enough literature on durability performance and what literature is available has conflicting results regarding chloride ion permeability, sulfate resistance, and carbonation.

Gayana	2018	[21]	Review Article	Concrete	IOT, FA, Coal, Recycled concrete, Crumb rubber, Glass, Plastic, Ceramic tile	Tailings with suitable admixtures as aggregates in concrete	-	More research on the combination of iron ore mine waste and tailings in concrete is required. IOT said to increase strength and density, so additives such as perlite and rubber are recommended to reduce the density.
Saedi	2021	[19]	Review Article	Concrete	Mining	Admixtures to mitigate issues with use	-	Admixtures used to i.e. increase pump-ability, reduce w/c ratio, increase density, adjust hydration and setting time.
Liu	2020	[102]	Review Article	Shotcrete		Rheological properties of fresh concrete and its application on shotcrete	-	Review on the correlation between shotcrete and rheological properties. The Bingham and modified Bingham models are the most used and accepted models for wet-mix shotcrete. Using rheological parameters, workability of shotcrete can be optimised.
Davoodi	2007	[60]	Primary Experimental Study	Concrete	Bauxite	Chemical and physical characterization of coarse bauxite residue (red sand) >100 mm for concrete making	w/c 0.35	Tailing processed and tested. Concrete mixtures included 1 - control, 2 - processed high silica red sand (HSRS), 3 - washed and carbonated red sand (WCRS), 4 - 1:1 ratio HSRS and WCRS, 5 - Red Mud (RM-fine), 6 - 1:1 ratio HSRS and RM, 7 - 1:1 ratio WCRS and RM, 8 - 0.9:0.1 ratio HSRS and RM, 9 - 0.9:0.1 WCRS and RM. From sieve

								analysis, both WCRS and HSRS were reasonably well graded and met the grading requirement as per AS 2758.1-1998, unfortunately, due to the high level of fines (<150 mm) for both types of red sands led to decrease workability higher water absorption from higher surface area and angular particle shape. 10% Red Mud demonstrated an increase in strength of 15% in the early ages and 5-10% for 28 days. 50-100% Red Mud as fine aggregate significantly reduces the workability and compressive strength of concrete. Mix 3,4,8,9 increased compressive strength by 7-16%, although HSRS declined on average 6% for all ages it still met the target strength of 50MPa. All subs led to decrease in tensile strength; best performing was WCRS with a 4% reduction compared to the reference. 10% RM led to a 25% decrease in tensile strength attributed to porous structure of red sand. All subs led to significant flexural strength due to the angular shape of red mud and red sand causing increased bonding between cement and fine aggregate. All sub except 10% red mud demonstrated poor durability (from low slump resulting in increased pores enabling water ingress, all others were marginal. Bauxite tailings cannot completely replace

								natural sand as it reduces strength dramatically. It was recommended that mix 4, HSRS and WCRS can be used in medium strength mixtures.
Salim	2023	[24]	Review Article	Concrete	Bauxite	Bauxite as a binding material, sub for cement	-	10% to 20% substantially enhances both mechanical and durability. Satisfactory performance against water absorption and corrosion potential is 10% - 20%, Rapid Chloride Penetration Test (RCPT) and sulfate resistance 10%-15%, sorptivity and electrical resistivity recommended 10% and 20%-30% respectively. Recommendations (further research) to assess the feasibility of incorporating fibers into cementitious composites to enhance tensile strength, additional research on durability (drying shrinkage, alkali-aggregate reaction, freeze and thaw, and creep), research focusing on mix design optimization, the effects of different methodologies (thermal activation or alkali activation to enhance pozzolanic activity.
Danso	2019	[61]	Primary Experimental Study	Concrete	Bauxite	Bauxite tailings to replace sand in concrete production 0, 25, 50, 75, 100%	w/c 0.6 Mix ratio 1:2:4	As the replacement value increases led to a significant increase in compressive, tensile, and flexural strengths compared to the control. 100% obtained the highest strength properties. Further research is required on the durability and chemical properties of

								concrete produced with bauxite mining waste.
Patangia	2023	[62]	Review Article	Concrete	Bauxite	Utilization of bauxite tailings as a supplementary cementitious material	-	Depending on the source, 10-20% can effectively replace cement. Bauxite mining waste (Red Mud) has roughly equal pozzolanic activity to that of FA, it contains many active elements, and its internal curing ability improves compressive strength. Conflicting results for setting time, however, RM was noted to generally increase setting time, variations in setting time were attributed to different particle size distributions and physical properties of RM and cement used. Slump decreases, due to particles being finer thus having a large surface area per kg and having many cracks and pores on their surface allows a greater absorption rate of water compared to cement. Conflicting optimal replacement levels are reported for compressive strength; values range from 5% to 25%. Heat-treated RM demonstrates enhanced pozzolanic and chemical activity and higher availability of SiO_2, TiO_2, and Al_2O_3. Tensile and flexural strength increased up to 12.5% for one study and was attributed to the fine particle size, its ability to act as a filler minimising micro-cracks, and the alkaline nature which

								promotes the formation of CSH gel. Durability - Utilising RM can decrease chloride penetration with the addition of coal refuse and FA, increase resistance to sulphate attack, carbonation decreases, alkali-silica reaction (ASR) reduces, and it improves resistance to freeze and thaw, due to micro filler effect and fine particle size. With increased RM leads to poor compressive strength performance as the number of fine particles in the interfacial transition zone (ITZ) weakens the bond between aggregate and hardened cement paste. Leaching results were found to be below the EPA limit for hazardous elements, even though RM leads to an increase in leaching chromium, cadmium, zinc, nickel, copper and lead.
Shirodkar	2023	[63]	Primary Experimental Study	Concrete	Bauxite	Investigated durability of concrete 0%, 5%, 10%, 15%, 20%, 25%, and 30% replacements. M30 grade concrete.	-	10% demonstrated better performance than the control. The 10% mix did not deteriorate from sulfate attack and saturation water absorption was reduced. With RM content above 10%, the compressive strength generally decreases.

Yalley	2022	[64]	Primary Experimental Study	Concrete	Bauxite	Bauxite tailings replaced cement at 10%, 15%, 20%, 25%, and 30%.	0.55	Compressive, flexural and corrosion permeability resistance properties were evaluated. Optimal ≤20%,
Zhou	2022	[65]	Primary Experimental Study	Concrete	Bauxite	The effect of thermal activation and particle size on cementitious activity of bauxite tailings	-	As the activation temperature increases, the particle size begins to decrease, however as the temperature exceeds 700°C, the particles increased in size due to the sintering process. The optimum activation temperature is 700°C and at this temperature with the increase in fineness, the activity index of the bauxite tailings increases. The fine tailings met the requirements to be used as an additive in Portland cement as it consumed more calcium hydroxide and the coarse tailings, which resulted in more C-S-H gel and ettringite.
Zhou	2023	[92]	Primary Experimental Study	3D Printing Mortar	Bauxite	Bauxite tailing sub for sand at 15%, 25%, 35%, and 45%		Optimal replacement for natural sand is 35%, obtaining good workability and high mechanical strength.
Zhou	2024	[93]	Primary Experimental Study	3D Printing Mortar	Bauxite	Sub for cement at 15%, 25%, 35%, and 45%	0.3	Optimal replacement for natural sand is 35%, compressive strength gains of 46.8% and flexural strength increase of 30.7% compared to the reference. Due to small size of the particles, active oxide in the tailings reacts

								with calcium hydroxide to generate a gel-like product making it dense compared to the control.
Ye	2017	[95]	Primary Experimental Study	Mortar	Bauxite and slag	Blended powder 70% calcined tailing and 30% slag, activated with 20% sodium silicate solution (water sub)	-	Slag powder and calcined bauxite tailing with a sodium silicate solution demonstrated steady strength increase from 50MPa at 28 days to 75 MPa at 6 years. Attributed to becoming more compact with age due to progressive geoplymerisation to form the coexisted C-A-S-H and N-A-S-H gels. C-A-S-H (Calcium Silicate Hydrate), N-A-S-H (Sodium Aluminosilicate Hydrate). The slag containing SiO_2 (32.6%), Al_2O_3 (29.9%) and CaO (13.6%) was thought to may have caused the formation of C-A-S-H gels in the hardened pastes.
Wang	2016	[94]	Primary Experimental Study	Mortar	Bauxite	Red Mud based field road cement, 0% and 23%.	-	RM did not affect the mineralogical properties of the prepared clinker, raw materials were subject to high heat treatment 1400°C for 30 min, compressive strength at early age is lower than the control, however at 28 days is higher due to C_4AF in the experimental mix (23% RM sub for cement) having a lower Al/Fe ratio which slows the hydration rate in early days.

Kumar	2022	[66]	Primary Experimental Study	Concrete	FA and Bauxite	FA 0-24% sub for cement, RM 0-24% with 8% FA subs for cement		Examined the use of two scenarios, RM in the presence of FA, and FA as a pozzolanic cementitious material. The mechanical strength of the concrete for all mixes performed better than the control. The optimal strength mix was 8% FA with 8% RM, then mix 16% FA. The optimal mixes for utilizing the greatest use of waste material were 24% RM with 8% FA, then mix 24% FA. No research was performed investigating later age strength or durability aspects of the substitutions.
Chavan	2021	[67]	Primary Experimental Study	Concrete	Bauxite	Bauxite (Red Mud, RM) substituting cement at 2, 4, 6, 8, 10, 12, 14, 16, 18, and 20%. M30 grade concrete	0.44	Slump decreased as content increased, Optimum substitution rate was 12% which demonstrated an increase in compressive, tensile, and flexural strength of 16.16%, 41.45% and 18.58% compared to the control. This was attributed to RM ability to enhance the internal microstructure resulting in concrete that is denser and has less voids, thus 12% sub also had the greatest decrease in chloride penetration which can mitigate the rate of corrosion of reinforcement.
Venkatesh	2019	[69]	Primary Experimental Study	Concrete	Bauxite	RM partially replaced cement from 0% to 15% at intervals of 2.5%.	0.4	The untreated RM was dried at 105°C for 24 h and the treated RM was given thermal treatment at 600°C for 2 h which enhanced the chemical and pozzolanic properties. Results indicated that the highest

						Comparing the effects of treated and untreated RM on the mechanical and durability properties of concrete.		compressive, tensile and flexural strength was achieved with treated RM at 10% and untreated RM at 5%. Interestingly from the data presented in the study suggests that RM treated and untreated at 0% demonstrated negligible differences in compressive strength and the same can be seen for treated and untreated RM at 5%. Regarding both tensile and flexural strength, the untreated RM demonstrated higher strength compared to the treated RM at 5%. Little difference is observed between the treated and untreated RM regarding chloride penetration and water absorption. As the RM content increased resulted in a decrease in chloride penetration and water absorption.
Venkatesh	2020	[68]	Primary Experimental Study	Concrete	Bauxite	RM partially replaced cement from 0%, 5%, 10%, 15% and 20%. Using pre-calcined (600°C for 2 hours) RM. Two scenarios with the variations in RM substitutions, A) Standard Grade concrete, B)	Mix A 0.4 w/c Mix B 0.38 w/c	Optimal strength was observed at a replacement of 10%. Mix A utilised 2.15 kg of superplasticiser, mix B utilised 4.3 kg of superplasticiser. Due to the RM containing high levels of alumina, this accelerated the cement hydration which was attributed to achieving high strength at an early age compared to the control. RM contributed to greater strength from an accelerated pozzolanic reaction between the cementitious materials due to the high alkaline (pH > 12) nature of the material. The strength increase at 10% replacement was also attributed to the

						High strength grade concrete. Mix B contained silica fume to achieve the higher strength.		higher C-S-H gel formation due to a high percentage of larnite, hatrurite, and low Ca/Si ratio. Larnite and hatrurite are both calcium silicate minerals that contribute to the formation of calcium silicate hydrate (C-S-H) gel and the low Ca/Si ratio enhances early curing age strength and contributes to stable C-S-H gel formation.

References

1. Ferdous, W.; Manalo, A.; Siddique, R.; Mendis, P.; Zhuge, Y.; Wong, H.S.; Lokuge, W.; Aravinthan, T.; Schubel, P. Recycling of landfill wastes (tyres, plastics and glass) in construction – A review on global waste generation, performance, application and future opportunities. *Resour. Conserv. Recycl.* **2021**, *173*, 105745.
2. Pickin, J.C.W.; O'Farrell, K.; Stovell, L.; Nyunt, P.; Guazzo, S.; Lin, Y.; Caggiati-Shortell, G.; Chakma, P.; Edwards, C.; Lindley, B.; et al. *National Waste Report 2022*; The Department of Climate Change, Energy, the Environment and Water: Docklands, VIC, Australia, 2022.
3. Nationwide Waste Solutions. 6 Facts You Should Know About Recycling Glass. Availabe online: https://nationwidewaste.com.au/6-facts-you-should-know-about-recycling-glass/ (accessed on 18 October 2023).
4. Victoria State Government. *National Approach to Manage Solar Panel, Inverter and Battery Life Cycles*; Victoria State Government: Melbourne, VIC, Australia, 2023.
5. Lambert, I. *Potential Impacts and Opportunities Arising from Australia's Green Energy Transition with Emphasis on Critical Minerals and Mining Wastes*; Geoscience Australia: Canberra, ACT, Australia, 2022.
6. Barcelos, D.A.; Pontes, F.V.M.; da Silva, F.A.N.G.; Castro, D.C.; dos Anjos, N.O.A.; Castilhos, Z.C. Gold mining tailing: Environmental availability of metals and human health risk assessment. *J. Hazard. Mater.* **2020**, *397*, 122721.
7. Australian Government. *Tailings Management—Leading Practice Sustainable Development Program for the Mining Industry*; Australian Government, Ed.; Australian Government: Canberra, ACT, Australia, 2016.
8. Csiro. Environmental Management for Mine Sites. Availabe online: https://www.csiro.au/en/work-with-us/industries/mining-resources?Social-and-environmental-performance?Minesite-environmental-managment (accessed on 27 June 2024).
9. Concrete Institute of Australia. *Recommended Practice—Shotcreting in Australia*; Concrete Institute of Australia: Crows Nest, NSW, Australia, 2010.
10. Yoggy, G.D. The history of shotcrete. *Shotcrete Am. Shotcrete Assoc.* **2000**, *2*, 28–29.
11. UNEP. *Sand and Sustainability: 10 Strategic Recommendations to Avert a Crisis*; GRID-Geneva, United Nations Environment Programme: Geneva, Switzerland, 2022.
12. UN Warns of Looming Sand Crisis as Population and City Growth Lead to Surging Demand. *ABC News*, 28 April 2022.
13. Budinski, K.; Budinski, M. *Engineering Materials: Properties and Selection*, 9th ed.; Pearson: Richmond, VIC, Australia, 2010.
14. Foster, S.; Kilpatrick, A.; Warner, R. *Reinforced Concrete Basics: Analysis and Design of Reinforced Concrete Structures*, 3rd ed.; Pearson: Richmond, VIC, Australia, 2021.
15. Woodson, R.D. *Concrete Portable Handbook*, 1st ed.; Butterworth-Heinemann: Amsterdam, The Netherlands, 2012.
16. Aïtcin, P.-C.; Flatt, R.J. *Science and Technology of Concrete Admixtures*; Elsevier Science & Technology: London, UK, 2015.
17. Rentier, E.S.; Cammeraat, L.H. The environmental impacts of river sand mining. *Sci. Total Environ.* **2022**, *838*, 155877.
18. Panesar, D.K.; Zhang, R. Performance comparison of cement replacing materials in concrete: Limestone fillers and supplementary cementing materials—A review. *Constr. Build. Mater.* **2020**, *251*, 118866.
19. Saedi, A.; Jamshidi-Zanjani, A.; Darban, A.K. A review of additives used in the cemented paste tailings: Environmental aspects and application. *J. Environ. Manag.* **2021**, *289*, 112501.
20. Gou, M.; Zhou, L.; Then, N.W.Y. Utilization of tailings in cement and concrete: A review. *Sci. Eng. Compos. Mater.* **2019**, *26*, 449–464.
21. Gayana, B.C.; Karra, R.C. Sustainable use of mine waste and tailings with suitable admixture as aggregates in concrete pavements-A review. *Adv. Concr. Constr.* **2018**, *6*, 221–243.

22. Ikotun, J.; Adeyeye, R.; Otieno, M. Application of mine tailings sand as construction material—A review. *MATEC Web Conf.* **2022**, *364*, 1–6.
23. Kazmi, D.; Williams, D.; Serati, M. Waste glass in civil engineering applications—A review. *Int. J. Appl. Ceram. Technol.* **2020**, *17*, 529–554.
24. Salim, M.U.; Mosaberpanah, M.A.; Danish, A.; Ahmad, N.; Khalid, R.A.; Moro, C. Role of bauxite residue as a binding material and its effect on engineering properties of cementitious Composites: A review. *Constr. Build. Mater.* **2023**, *409*, 133844.
25. Sathiparan, N.; Subramaniam, D.N. Potential use of crushed waste glass and glass powder in sustainable pervious concrete: A review. *Clean. Waste Syst.* **2024**, *9*, 100191.
26. Kazmi, D.; Serati, M.; Williams, D.J.; Qasim, S.; Cheng, Y.P. The potential use of crushed waste glass as a sustainable alternative to natural and manufactured sand in geotechnical applications. *J. Clean. Prod.* **2021**, *284*, 124762.
27. Steyn, Z.C.; Babafemi, A.J.; Fataar, H.; Combrinck, R. Concrete containing waste recycled glass, plastic and rubber as sand replacement. *Constr. Build. Mater.* **2021**, *269*, 121242.
28. Tamanna, N.; Tuladhar, R.; Sivakugan, N. Performance of recycled waste glass sand as partial replacement of sand in concrete. *Constr. Build. Mater.* **2020**, *239*, 117804.
29. Win, T.K.; Pauzi, N.I.M. Investigation of the Optimum Shotcrete Mixing Ratio for Slope Protection using Fly Ash. *MATEC Web Conf.* **2023**, *384*, 02004.
30. Wang, Q. Study on the effect of fineness and dosage of fly ash on mechanical properties of concrete. *IOP Conf. Ser. Earth Environ. Sci.* **2021**, *787*, 012009.
31. Wu, B.; Chai, M.; Li, S.; Yang, J. Effect of Fly Ash Content in Shotcrete on Mechanical Properties. *J. Phys. Conf. Ser.* **2022**, *2168*, 012013.
32. Lu, W.; Wang, J.; Peng, Z.; Zhang, B. Research on influences of fly ash content on mechanical properties of shotcrete. *IOP Conf. Ser. Mater. Sci. Eng.* **2018**, *452*, 022063.
33. Sun, J.; Li, R.Y.M.; Li, L.; Deng, C.; Ma, S.; Zeng, L. Develop a sustainable wet shotcrete for tunnel lining using industrial waste: A field experiment and simulation approach. *Adv. Concr. Constr.* **2023**, *15*, 333–348.
34. Australian Government. *Atlas of Australian Mine Waste*; Australian Government—Geoscience Australia: Canberra, ACT, Australia, 2024.
35. Ishaq, M.B.; Mohammed, A.S.; Mohammed, A.A. Novel exploration of chemical compounds in mono-variate, Bi-variate, and multi-variate scenarios of waste glass powder as a cement replacement in concrete. *Sustain. Chem. Pharm.* **2025**, *43*, 101894.
36. Jahami, A.; Khatib, J.; Raydan, R. Production of Low-Cost, High-Strength Concrete with Waste Glass as Fine Aggregates Replacement. *Buildings* **2022**, *12*, 2168. https://doi.org/10.3390/buildings12122168.
37. Jiao, Y.; Zhang, Y.; Guo, M.; Zhang, L.; Ning, H.; Liu, S. Mechanical and fracture properties of ultra-high performance concrete (UHPC) containing waste glass sand as partial replacement material. *J. Clean. Prod.* **2020**, *277*, 123501.
38. Wei, L.; Zhang, N.; Sun, L. Rebar corrosion and bond in concrete with recycled glass granules. *J. Build. Eng.* **2024**, *98*, 111269.
39. Bhat, K.R.; Dumre, G.; Gyawali, T.R. Transforming waste into strength: Evaluating properties of concrete with waste glass substitution. *Clean. Waste Syst.* **2024**, *9*, 100179.
40. Adaway, M.; Wang, Y. Recycled glass as a partial replacement for fine aggregate in structural concrete—Effects on compressive strength. *Electron. J. Struct. Eng.* **2015**, *14*, 116–122.
41. Mohammed, T.K.; Hama, S.M. Mechanical properties, impact resistance and bond strength of green concrete incorporating waste glass powder and waste fine plastic aggregate. *Innov. Infrastruct. Solut.* **2021**, *7*, 49.
42. Al-Kizwini, R.S. Reuse plastic and glass wastes as a partial replacement of concrete components. *J. Eng. Sci. Technol.* **2020**, *15*, 1109–1123.

43. Song, J.; Peng, L.; Zhao, Y. Synergetic valorization of recycled aggregates and waste glass powder in sustainable concrete: Microstructure, mechanical strength, and bonding performance. *Constr. Build. Mater.* **2025**, *458*, 139609.
44. Gerges, N.N.; Issa, C.A.; Fawaz, S.A.; Jabbour, J.; Jreige, J.; Yacoub, A. Recycled Glass Concrete: Coarse and Fine Aggregates. *Eur. J. Eng. Technol. Res.* **2018**, *3*, 1–9.
45. Rahma, A.; El Naber, N.; Issa Ismail, S. Effect of glass powder on the compression strength and the workability of concrete. *Cogent Eng.* **2017**, *4*, 1373415.
46. Huang, X.; Huang, J.; Kaewunruen, S. An explainable machine learning system for efficient use of waste glasses in durable concrete to maximise carbon credits towards net zero emissions. *Waste Manag.* **2025**, *193*, 539–550.
47. Disfani, M.M.; Arulrajah, A.; Bo, M.W.; Sivakugan, N. Environmental risks of using recycled crushed glass in road applications. *J. Clean. Prod.* **2012**, *20*, 170–179.
48. Ali, E.E.; Al-Tersawy, S.H. Recycled glass as a partial replacement for fine aggregate in self compacting concrete. *Constr. Build. Mater.* **2012**, *35*, 785–791.
49. Ahmad, S.; Upadhyay, S.; Umar, A.; Al-Osta, M.A. Effect of recycled crushed glass and recycled coarse aggregate on the properties of self-compacting concrete. *Case Stud. Constr. Mater.* **2023**, *19*, e02532.
50. Ismail, Z.Z.; Al-Hashmi, E.A. Recycling of waste glass as a partial replacement for fine aggregate in concrete. *Waste Manag.* **2009**, *29*, 655–659.
51. Basireddy, S.M.R.; Serati, M.; Jackson, N.; Malgotra, G.; Asche, H. Utilization of crushed waste glass in shotcrete. In *ARMA US Rock Mechanics/Geomechanics Symposium*; ARMA: Eaton, CO, USA, 2021.
52. Serati, M.; Jakson, N.; Asche, H.; Basireddy, S.; Malgotra, G. Sustainable shotcrete production with waste glass aggregates. *SN Appl. Sci.* **2022**, *4*, 82.
53. Augonis, A.; Ivanauskas, E.; Bocullo, V.; Kantautas, A.; Vaičiukynienė, D. The Influence of Expanded Glass and Expanded Clay on Lightweight Aggregate Shotcrete Properties. *Materials* **2022**, *15*, 1674.
54. Wang, T.; Nicolas, R.S.; Kashani, A.; Ngo, T. Sustainable utilisation of low-grade and contaminated waste glass fines as a partial sand replacement in structural concrete. *Case Stud. Constr. Mater.* **2022**, *16*, e00794.
55. Liu, X.; Ye, Z.; Lu, J.-X.; Xu, S.; Hsu, S.-C.; Poon, C.S. Comparative LCA-MCDA of high-strength eco-pervious concrete by using recycled waste glass materials. *J. Clean. Prod.* **2024**, *479*, 144048.
56. Asa, E.; Anna, A.S.; Baffoe-Twum, E. An investigation of mechanical behavior of concrete containing crushed waste glass. *J. Eng. Des. Technol.* **2019**, *17*, 1285–1303.
57. Song, H.; Ahmad, A.; Farooq, F.; Ostrowski, K.A.; Maślak, M.; Czarnecki, S.; Aslam, F. Predicting the compressive strength of concrete with fly ash admixture using machine learning algorithms. *Constr. Build. Mater.* **2021**, *308*, 125021.
58. Reshma, T.V.; Manjunatha, M.; Sankalpasri, S.; Tanu, H.M. Effect of waste foundry sand and fly ash on mechanical and fresh properties of concrete. *Mater. Today Proc.* **2021**, *47*, 3625–3632.
59. Zou, X.; Zhang, Y.; Liu, B.; Xu, X.; Lu, Z.; Jiang, W. Study on mechanical properties and ratio parameter optimization of fly ash wet shotcrete under the influence of multiple factors. *Case Stud. Constr. Mater.* **2023**, *19*, e02679.
60. Davoodi, M.; Nikraz, H.; Jamieson, E. Chemical and physical characterization of coarse bauxite residue (red sand) for concrete making. In *Sustainable Construction Materials and Technologies*; Taylor & Francis Group: London, UK, 2007.
61. Danso, H.; Boadi, J.K. Replacement of Sand with Bauxite Mining Waste in Concrete Production. *J. Mater. Eng. Struct.* **2019**, *6*, 525–534.
62. Patangia, J.; Saravanan, T.J.; Kabeer, K.I.S.A.; Bisht, K. Study on the utilization of red mud (bauxite waste) as a supplementary cementitious material: Pathway to attaining sustainable development goals. *Constr. Build. Mater.* **2023**, *375*, 131005.
63. Shirodkar, V.; Patel, R.; Verma, M.; Mahate, V. An investigation into the durability of concrete using red mud as a substitute in cement. *Mater. Today Proc.* **2023**. https://doi.org/10.1016/j.matpr.2023.12.010.

64. Yalley, P.P.; Kankam, C.K. Compressive, flexural and corrosion permeability resistance properties of concrete with bauxite tailing as supplementary mineral admixtures. *Sci. Afr.* **2022**, *18*, e01409.
65. Zhou, L.; Gou, M.; Hou, W.; Zhao, M.; Zhao, J.; Shen, Z. Effect of thermal activation and particle size on cementitious activity of bauxite tailings. *Environ. Sci. Pollut. Res.* **2022**, *29*, 78960–78972.
66. Kumar, K.; Bansal, M.; Garg, R.; Garg, R. Mechanical strength analysis of fly-ash based concrete in presence of red mud. *Mater. Today Proc.* **2022**, *52*, 472–476.
67. Chavan, S.P.; Salokhe, S.A.; Nadagauda, P.A.; Patil, S.T.; Mane, K.M. An investigational study on properties of concrete produced with industrial waste red mud. *Mater. Today Proc.* **2021**, *42*, 733–738.
68. Venkatesh, C.; Nerella, R.; Chand, M.S.R. Role of red mud as a cementing material in concrete: A comprehensive study on durability behavior. *Innov. Infrastruct. Solut.* **2020**, *6*, 13.
69. Venkatesh, C.; Ruben, N.; Madduru, S.R. Comparison of mechanical and durability properties of treated and untreated red mud concrete. *Mater. Today Proc.* **2019**, *27*, 284–287.
70. Zhang, W.; Gu, X.; Qiu, J.; Liu, J.; Zhao, Y.; Li, X. Effects of iron ore tailings on the compressive strength and permeability of ultra-high performance concrete. *Constr. Build. Mater.* **2020**, *260*, 119917.
71. Shi, J.; Pan, W.; Kang, J.; Yu, Z.; Sun, G.; Li, J.; Zheng, K.; Shen, J. Properties of Ultra-High Performance Concrete incorporating iron tailings powder and iron tailings sand. *J. Build. Eng.* **2024**, *83*, 108442.
72. Liang, H.; Liu, J.; Kang, B.; Zhang, Y.; He, Y.; Yang, H. Optimizing 1-hydroxyethylidene-1,1-diphosphonic acid and mechanically-activated iron ore tailings to prepare low-cost and retarded magnesium oxysulfate cement. *Case Stud. Constr. Mater.* **2022**, *17*, e01631.
73. Lv, X.; Shen, W.; Wang, L.; Dong, Y.; Zhang, J.; Xie, Z. A comparative study on the practical utilization of iron tailings as a complete replacement of normal aggregates in dam concrete with different gradation. *J. Clean. Prod.* **2019**, *211*, 704–715.
74. Yang, M.; Sun, J.; Dun, C.; Duan, Y.; Meng, Z. Cementitious activity optimization studies of iron tailings powder as a concrete admixture. *Constr. Build. Mater.* **2020**, *265*, 120760.
75. Jayasimha, N.; Sujini, B.; Annapurna, B.P. A study on durability and strength properties of high strength concrete with partial replacement of iron ore tailings with fine aggregates. *Mater. Today Proc.* **2022**, *65*, 1922–1929.
76. Vescovi, P.H.T.; Bezerra, A.C.d.S.; Poggiali, F.S.J. Iron ore tailing (IOT) chemical composition and content impact on the physical, mechanical, and durability properties of blended cement. *J. Build. Eng.* **2023**, *80*, 108119.
77. Han, F.; Zhang, H.; Liu, J.; Song, S. Influence of iron tailing powder on properties of concrete with fly ash. *Powder Technol.* **2022**, *398*, 117132.
78. Goulart Bezerra, C.; Abelha Rocha, C.A.; Siqueira, I.S.d.; Toledo Filho, R.D. Feasibility of iron-rich ore tailing as supplementary cementitious material in cement pastes. *Constr. Build. Mater.* **2021**, *303*, 124496.
79. Cheng, Y.; Huang, F.; Qi, S.; Li, W.; Liu, R.; Li, G. Durability of concrete incorporated with siliceous iron tailings. *Constr. Build. Mater.* **2020**, *242*, 118147.
80. Ling, J.; Wang, K.; Wang, M.; Tao, Y.; Wu, T. Damage evaluation of concrete using iron ore tailings as aggregates under uniaxial cyclic compression. *Granul. Matter* **2023**, *25*, 25.
81. Fan, Y.y.; Wang, X.; Jiang, M.-w.; Liu, X.-q. The microstructure and mechanical property of tailings and its application in artificial concrete. *Arab. J. Geosci.* **2021**, *14*, 2105.
82. Song, Q.; Zou, Y.; Bao, J.; Zhang, P. Disposal of solid waste as building materials: A study on the mechanical and durability performance of concrete composed of gold tailings. *J. Mater. Res. Technol.* **2024**, *30*, 2111–2124.
83. Widojoko, L.; Hardjasaputra, H.; Susilowati. Study of Gold Mine Tailings Utilization as Fine Aggregate Material for Producing Shotcrete Based on Concept of Green Technology. In Proceedings of the 3rd International Conference on Engineering and Technology Development 2014, Bandar Lampung, Indonesia, 28–29 October 2014.
84. Luo, B.; Luo, Z. Influence of curing regimes on the mechanical properties and microstructure of ultra-high-performance concrete blended with gold mine tailings. *Mater. Lett.* **2022**, *311*, 131527.

85. Garcia-Troncoso, N.; Baykara, H.; Cornejo, M.H.; Riofrio, A.; Tinoco-Hidalgo, M.; Flores-Rada, J. Comparative mechanical properties of conventional concrete mixture and concrete incorporating mining tailings sands. *Case Stud. Constr. Mater.* **2022**, *16*, e01031.
86. Thomas, B.S.; Damare, A.; Gupta, R.C. Strength and durability characteristics of copper tailing concrete. *Constr. Build. Mater.* **2013**, *48*, 894–900.
87. Zhang, Z.; Gao, N.; Wang, H. The Effect of Copper Tailings Sand on the Workability and Mechanical Properties of Concrete. *Buildings* **2024**, *14*, 1789. https://doi.org/10.3390/buildings14061789.
88. Gutierrez, M.; Gomez, R.; Alcayaga, A.; Avudaiappan, S.; Betancourt, F. Potential of copper tailings as fine aggregates in cement mortar. In Proceedings of the 9th International Conference and Exhibition on Mass Mining, Kiruna Sweden, 17–19 September 2024.
89. Xiao, M.; Ju, F.; He, Z.-q. Research on shotcrete in mine using non-activated waste coal gangue aggregate. *J. Clean. Prod.* **2020**, *259*, 120810.
90. Sun, J.; Liu, S.; Ma, Z.; Qian, H.; Wang, Y.; Al-azzani, H.; Wang, X. Mechanical properties prediction of lightweight coal gangue shotcrete. *J. Build. Eng.* **2023**, *80*, 108088.
91. Qiu, J.; Zhu, M.; Zhou, Y.; Guan, X. Effect and mechanism of coal gangue concrete modification by fly ash. *Constr. Build. Mater.* **2021**, *294*, 123563.
92. Zhou, L.; Gou, M.; Zhang, H. Investigation on the applicability of bauxite tailings as fine aggregate to prepare 3D printing mortar. *Constr. Build. Mater.* **2023**, *364*, 129904.
93. Zhou, L.; Gou, M.; Ji, J.; Hou, X.; Zhang, H. Durability and hardened properties of 3D printed concrete containing bauxite tailings. *Mater. Today Sustain.* **2024**, *25*, 100704.
94. Wang, X.; Luo, Z.; Zhang, L.; Rong, H.; Yang, J. Utilization of red mud as raw material in the production of field road cement. *J. Wuhan Univ. Technol.-Mater. Sci. Ed.* **2016**, *31*, 877–882.
95. Ye, J.; Zhang, W.; Shi, D. Properties of an aged geopolymer synthesized from calcined ore-dressing tailing of bauxite and slag. *Cem. Concr. Res.* **2017**, *100*, 23–31.
96. Hind, A.R.; Bhargava, S.K.; Grocott, S.C. The surface chemistry of Bayer process solids: A review. *Colloids Surf. A Physicochem. Eng. Asp.* **1999**, *146*, 359–374.
97. Romano, R.C.d.O.; Bernardo, H.M.; Maciel, M.H.; Pileggi, R.G.; Cincotto, M.A. Using isothermal calorimetry, X-ray diffraction, thermogravimetry and FTIR to monitor the hydration reaction of Portland cements associated with red mud as a supplementary material. *J. Therm. Anal. Calorim.* **2019**, *137*, 1877–1890.
98. Romano, R.C.O.; Bernardo, H.M.; Maciel, M.H.; Pileggi, R.G.; Cincotto, M.A. Hydration of Portland cement with red mud as mineral addition. *J. Therm. Anal. Calorim.* **2018**, *131*, 2477–2490.
99. Senff, L.; Hotza, D.; Labrincha, J.A. Effect of red mud addition on the rheological behaviour and on hardened state characteristics of cement mortars. *Constr. Build. Mater.* **2011**, *25*, 163–170.
100. Sambangi, A.; Arunakanthi, E.J.M.T.P. Fresh and mechanical properties of SCC with fly ash and copper slag as mineral admixtures. *Mater. Today Proc.* **2021**, *45*, 6687–6693.
101. Yu, L.; Wang, S.; Qiao, Z.; Xu, L.; Wu, K.; Li, P.; Yang, Z. Effect of curing time and temperature on the mechanical properties of green and ultra-high-strength non-sintered aggregate via autoclave technology. *Constr. Build. Mater.* **2023**, *374*, 130874.
102. Liu, G.; Cheng, W.; Chen, L.; Pan, G.; Liu, Z. Rheological properties of fresh concrete and its application on shotcrete. *Constr. Build. Mater.* **2020**, *243*, 118180.
103. Thang, N.; Tuan, N.; Hiep, D.; Thang, V. The Potential Use of Waste Rock from Coal Mining for the Application as Recycled Aggregate in Concrete. In *Proceedings of the International Conference on Innovations for Sustainable and Responsible Mining: ISRM 2020-Volume 1*; Springer International Publishing: Cham, Switzerland, 2021; pp. 550–561.
